# Characterization of circSCL38A1 as a novel oncogene in bladder cancer via targeting ILF3/TGF-β2 signaling axis

**DOI:** 10.1038/s41419-023-05598-2

**Published:** 2023-01-25

**Authors:** Peilong Li, Qi Mi, Suzhen Yan, Yan Xie, Zilian Cui, Shujun Zhang, Yifan Wang, Huiru Gao, Yunshan Wang, Juan Li, Lutao Du, Chuanxin Wang

**Affiliations:** 1 Department of Clinical Laboratory, The Second Hospital of Shandong University, Jinan, Shandong 250033 China; 2 Shandong Engineering & Technology Research Center for Tumor Marker Detection, Jinan, Shandong China; 3 Department of Urology, Shandong Provincial Hospital, Shandong University, Jinan, Shandong 250021 China; 4 Shandong Provincial Clinical Medicine Research Center for Clinical Laboratory, Jinan, Shandong China; 5 Shandong Technology Innovation Center for Big Data and Precision Medicine of Cancer, Jinan, 250033 Shandong China

**Keywords:** Bladder cancer, Non-coding RNAs, Epithelial-mesenchymal transition, Tumour biomarkers

## Abstract

The regulatory role of circRNAs in cancer metastasis has become a focused issue in recent years. To date, however, the discovery of novel functional circRNAs and their regulatory mechanisms via binding with RBPs in bladder cancer (BC) are still lacking. Here, we screened out circSLC38A1 based on our sequencing data and followed validation with clinical tissue samples and cell lines. Functional assays showed that circSLC38A1 promoted BC cell invasion in vitro and lung metastasis of mice in vivo. By conducting RNA pull-down, mass spectrum, and RIP assays, circSLC38A1 was found to interact with Interleukin enhancer-binding factor 3 (ILF3), and stabilize ILF3 protein via modulating the ubiquitination process. By integrating our CUT&Tag-seq and RNA-seq data, TGF-β2 was identified as the functional target of the circSLC38A1-ILF3 complex. In addition, m6A methylation was enriched in circSLC38A1 and contributed to its upregulation. Clinically, circSLC38A1 was identified in serum exosomes of BC patients and could distinguish BC patients from healthy individuals with a diagnostic accuracy of 0.878. Thus, our study revealed an essential role and clinical significance of circSLC38A1 in BC via activating the transcription of TGF-β2 in an ILF3-dependent manner, extending the understanding of the importance of circRNA-mediated transcriptional regulation in BC metastasis.

## Introduction

In recent years, the incidence and mortality of Bladder cancer (BC) have risen and made it the highest-ranked carcinoma in the urinary system [1, 2]. Because of the lack of diagnostic approaches, approximately one-quarter of patients with BC were diagnosed as muscular invasive once found, resulting in a worse prognosis due to the development of distant metastases [3–5]. Although the effectiveness of clinical interventions for BC, such as surgical operation, radiation therapy, and chemotherapy, has been continuously improved in recent decades, the overall therapeutic effects for BC patients were still poor [6, 7]. Therefore, finding promising biomarkers and screening effective therapeutic targets have great significance for optimizing personalized treatment and improving the prognosis of BC patients.

Circular RNAs (circRNAs) are a group of novel transcripts whose 3′ and 5′ RNA ends are covalently bound to form a special loop structure [8–10]. Recently, research on the role of circRNAs in the pathogenesis and progression of cancers has been deepened, and the relationship between them has attracted more and more attention in the field of cancer research. Dysregulated circRNAs in cancers could regulate cellular functions in multiple ways [11–14]. A well-established post-transcriptional regulation mode for circRNAs is through sponging miRNAs to regulate the miRNA-related genes [15–18]. Moreover, a proportion of circRNAs could be translated and function to encode protein [19]. It is worth noting that a specific category of circRNAs could bind to RNA-binding proteins (RBPs), such as transcription factors, to form an RNA-protein complex and regulate the transcriptional activity of targeted functional genes [19, 20]. However, whether this regulatory mode of circRNAs applies in BC and the detailed mechanism are not revealed yet.

Interleukin enhancer-binding factor 3 (ILF3), also as termed as CCAAT box transcription factor (CBTF), is spliced from the ILF3 gene and plays essential roles via serving as double-stranded RBPs [21]. It was reported that ILF3 was up-regulated and acted as a catalyst in multiple types of malignancies [22–26]. In some cases, the upregulation of ILF3 plays an essential role in the reprogramming of systemic serine metabolism via the EGF-ERK signaling and confers a predilection towards colorectal cancer development [24]. Highly expressed ILF3 could upregulate sustained uPA and thereby promote breast tumorigenicity [26]. However, few studies focus on the influence of ILF3 in the development and potential mechanism of BC via the binding with circRNAs.

This work identified hsa_circ_0000396, which we renamed circSLC38A1 (a circular RNA derived from the SLC38A1 gene), critical for BC metastasis. Moreover, circSLC38A1 was upregulated in BC cells due to the enriched N6-methyladenosine (m6A) modification. Mechanistic studies revealed that circSLC38A1 could form a complex by interacting with ILF3 and stabilizing its stability, and this complex could finally initiate TGF-β2 expression according to our integrative analysis. Finally, circSLC38A1 can be packaged into exosomes and may be used as a potential diagnostic biomarker for BC patients.

## Materials and methods

### Clinical samples

A total of 3 cohorts of samples were enrolled in this study. Cohort I contained 41 pairs of BC tissues and adjacent noncancerous tissues (more than 3 cm between the two types of tissues in the same patient) obtained from BC patients who received surgical resection at The Second Hospital of Shandong University from January 2017 to February 2020. Five paired samples from cohort I were enrolled for RNA sequencing analysis, and the other 36 paired samples were for qRT-PCR verification. These tissues were obtained followed by immediately stored in liquid nitrogen. Cohort II containing 63 BC tissues and 16 noncancerous tissues with survival information was used for tissue microarray (TMA) to validate the in situ expression of circSLC38A1 in BC and noncancerous tissues.

Cohort III containing serum samples of 65 BC patients and 64 healthy people were collected from The Second Hospital of Shandong University from June 2019 to June 2021. Serum samples were centrifuged at 3000 × *g* for 15 min and then stored frozen at −80 °C until used. Serum exosomes were extracted with ExoQuick^TM^ Precipitation Solution (System Biosciences, PA, USA) following the user manual. The morphology of exosomes was observed with the electron microscope. The nanoparticle tracking analysis (NTA) was then performed using ZetaView (Particle Metrix, Germany) to detect isolated exosomes’ concentration and size distribution. Serum exosomes were also identified by detecting the expression of exosomal positive protein markers (CD9, CD63, and TSG101).

The inclusion and exclusion criteria were as follows: 1) Pathologically diagnosed as BC by at least two pathologists; 2) Patients didn’t receive radiotherapy or chemotherapy before surgery; 3) No other diseases that seriously threaten the patient’s life; 4) No malignant tumors other than BC. Permission from the Institutional Review Board of The Second Hospital of Shandong University was obtained (KYLL-2018(KJ)P-0024), and the informed consent from all participants was signed. The detailed information of patients in cohort II and cohort III were shown in Tables S1 and S2, respectively.

### Cell lines and cell culture

Normal human urothelial line (SV-HUC-1), five bladder transitional cell carcinoma cell lines (UMUC3, J82, T24, RT4, SW780), and Grade II bladder carcinoma cell line (5637) were obtained from American Type Culture Collection (ATCC, MD, USA). SV-HUC-1 cells were cultured in F-12 K medium (Gibco, Grand Island, NY, USA), UMUC3 and J82 cells were maintained in MEM medium (Gibco), T24 and RT4 cells were cultured in McCoy’s 5 A medium (Gibco), SW780 cells were cultured in L-15 medium (Gibco), and 5637 cells were cultured in RPMI-1640 medium (Gibco). All the mediums contain 1% penicillin/streptomycin (Gibco) and 10% fetal bovine serum (FBS) (Gibco). Cells were cultured in an incubator at 37 °C in a humidified atmosphere of 5% CO_2_.

### RNA sequencing analysis

Five paired BC and normal samples from cohort I were utilized for circRNA high throughput sequence analysis. The 5 paired samples used for sequencing analysis were selected with those guidelines: 1) size of the tumor and normal area were more than 5 grams; 2) tumor tissues with low heterogeneity and the normal parts with over 80% epithelial tissue; 3) patient with BC at NMIBC (stage I) and MIBC (II). Detailed information on the 5 patients is shown in Table S3. Total RNA was isolated with a Trizol reagent (Invitrogen, Carlsbad, CA, USA). The RNA concentration was detected using a NanoDrop 2000 instrument (NanoDrop Technologies, Wilmington, DE, USA). The RNA integrity was assessed by Agilent 2100 Bioanalyzer (Asiagen Technology, Shanghai, China). Following the vendor’s recommended protocol, the circRNA sequencing was performed on an Illumina Hiseq 4000 (LC Bio, Shanghai, China). The significantly upregulated or downregulated circRNAs were screened out with the expression fold change >2 and < −2, respectively. *P* < 0.05 rule was set as statistical significance (R package-edgeR). The GEO Accession number is GSE190079.

For exploring differentially expressed genes potentially targeted by circSLC38A1, the sequencing experiments were also performed in T24 cells transfected with si-NC and si-circSLC38A1 (Jiayin Biomedical Technology, Shanghai, China), and target genes were analyzed. The dysregulated RNAs were screened by R package–Deseq2 with the standard as described above. The GEO Accession number is GSE186226.

### Animal treatments

All experiments were approved by the Animal Care and Use Committee of The Second Hospital of Shandong University (Animal Protocol Number: KYLL-2018(KJ)A-0028). To explore the potential roles of circSLC38A1 in the metastasis of BC, a total of 20 4-week-old BALB/c male nude mice were purchased from the Experimental Animal Center of Shandong University. Mice were blindingly and randomly allocated into two groups and raised under pathogen-free conditions. Cells were harvested and suspended in PBS on ice (1 × 10^6^ per mouse/100 μl) and were then injected into mice through the tail vein. Mice were sacrificed after injection for 8 weeks, and the IVIS spectrum CT (PerkinElmer, Shanghai, China) was applied to generate xenograft images with fluorescence signals. A total of 5 and 7 mice were obtained in the control and shRNA groups, respectively.

### RNA and gDNA isolation

Total RNAs were extracted and purified with a Trizol-based method according to the manufacturer’s instructions (Invitrogen). In contrast, Exosome Isolation Kit (EZBioscience, Shanghai, China) was used to extract and purified exosomal RNAs. Genomic DNA Isolation Kit (TIANGEN Biotech, Beijing, China) was used to isolate gDNA of BC cells. The intensity and quality of the extracted RNAs and gDNA were evaluated with a NanoDrop2000 instrument (NanoDrop Technologies).

### quantitative real-time polymerase chain reaction (qRT-PCR)

cDNA was synthesized using PrimeScript^TM^ RT reagent kit (Takara Bio Inc. Dalian, China) with 1 µg RNA as a template on T100^TM^ Thermal Cycler (Bio-Rad, Shanghai, China). qRT-PCR was carried out using TB Green Premix Ex Taq ^TM^ reagent kit (Takara) on a CFX96^TM^ Real-Time PCR System (Bio-Rad) with 1 µl cDNA/gDNA as a template. The products of qRT-PCR were verified by conducting 2% agarose gel electrophoresis. GAPDH was used as the internal reference. Relative expression levels of circRNAs and mRNAs were normalized to GAPDH and calculated by the 2^−ΔΔCt^ method. The details of primers are listed in Table S4.

### Chemical treatments

DNA methyltransferase inhibitor 5-aza-dC (MedChemExpress, Shanghai, China) was used at the concentration of 5 µM for 7 days when treating BC cells. Broad-spectrum histone deacetylase (HDAC) inhibitors, SAHA and NAB ((MedChemExpress), were used at the concentration of 2 µM for 24 h. Actinomycin D (ActD) (Sigma, USA) was used at the concentration of 2 μg/ml for several time periods. Total RNA (2 μg) extracted from T24 was incubated with 3 U/μg of RNase R (Epicentre Technologies, Madison, USA) for 15 min at 37 °C. The expression levels of circSLC38A1 and other mRNAs were analyzed by qRT-PCR.

### Plasmid construction

The pLCDH-ciR vector and circSLC38A1 overexpression plasmid were synthesized from Geneseed Biotech Co (Guangzhou, China). Overexpression plasmids of HA-ubi, METTL3, FTO and Flag-ILF3 WT plasmid were obtained from VigeneBio (Shangdong, China). The plasmid transfection process was operated by using Lipofectamine 3000 (Invitrogen) as per the product guidelines.

### RNA interference (RNAi) and transfection

siRNAs duplexes for silencing circSLC38A1, ILF3, METTL3, and FTO were generated by GenePharma (Shanghai, China). The circSLC38A1 silencing lentivirus was synthesized in GenePharma by loading a short hairpin circRNA-SLC38A1 sequence (according to si-circSLC38A1#2) into LV3. The negatively controlled silencing vector was the same vector of shcirc to eliminate the influence of vectors. In addition, the sequence for negative control was designed as non-homologous to silencing sequences. This control sequence was tested by running blast in the genome from human, Norway rat and house mouse and no matching sequences were obtained. For in vitro cell transfection, Lipofectamine 3000 was used to dysregulate respective transcripts in BC cells. Related sequences information was listed in Table S5.

### Western blot assay

RIPA buffer (Beyotime Biotechnology, Shanghai, China) was used to lysis cells and isolated proteins. Extracted proteins were then quantified with a BCA Protein assay kit (Beyotime Biotechnology) according to the product specification. The proteins were separated with electrophoresis, then transferred from the gel to a polyvinylidene difluoride (PVDF) membrane using the semi-dry-transfer method. After transfer, the membrane was washed with TBST buffer twice and blocked at 25 °C with 5% defatted milk powder for 1 h, and the membrane was washed with TBST three times and put into a sealed bag containing the primary antibody solution (1:1000) and incubated overnight at 4 °C. Next day, the membranes were hybridized with specific HRP-conjugated secondary antibodies at ~25°C for 1 h with a dilution ratio of 1:5000. Imagines were captured by using ChemiDoc^TM^ MP Imaging System (Bio-Rad). Primary antibodies used in this study include Vimentin (5741 S, CST), Snail (3879 S, CST), ILF3 (ab133354, Abcam), METTL3 (96391, CST), FTO (31687 S, CST), HA-tag (3724 S, CST), TGF beta 2 (36495, Abcam), N-cadherin (13116 S, CST), CD9 (13403, CST), CD63 (ab134045, Abcam), E-cadherin (3195 S, CST), TSG101 (ab125011, Abcam) and anti-GAPDH (5174 S, CST). Non-specific bands may exist to mix the visualization of ILF3, and all experimental operations will be performed accurately to quantify correctly.

### Fluorescence in situ hybridization (FISH)

CircSLC38A1-specific probe was designed and synthesized by GenePharma. T24 cells were seeded in 24-well plates covered with autoclaved glass slides at the bottom. After being cultured overnight, cells attached to slides were fixed followed by washing with PBS thoroughly. Then, cells were permeabilized with Triton, and incubated with 100 μl 2 × SSC at 37 °C for 30 min. Then hybridization was carried out with 2 μM probes specific to circSLC38A and U6 at 37 °C for 12 h under a dark environment. Finally, the slices were sealed with parafilm containing DAPI after gradually washing with 2×/1×SSC preheated to 42 °C. The images were captured using a Zeiss ZFM-700 fluorescence microscopy (Heidenheim, Germany). The probe sequence was shown as below:

circSLC38A1: 5’-ACACTCAATAAATATAGAAAAAGTAGGAT -3’

18 s: 5’- CTGCCTTCCTTGGATGTGGTAGCCGTTTC -3’

### RNA detection of TMA tissues

Human BC tissue microarray (TMA) that consisted of BC tissues (*n* = 63) and noncancerous tissues (*n* = 19) were purchased from Outdo Biotech, Ltd. (Shanghai, China). The tissues were firstly dewaxed at a temperature of 60 °C for 1 h, followed by immersion with xylene and ethanol. After washing with PBS, TMA was then digested with proteinase K for 20 min at room temperature. Pre-hybrid was then performed at 40 °C for 2 h. After the circSLC38A1-specific probe was used for hybridization at 40 °C overnight, followed by culture with biotinylated mouse anti-digoxin after washing with SSC. Finally, the tissues were visualized with NBT staining and nuclear fast red counterstain. The staining score was calculated based on the intensity (no intensity:0; weak intensity:1 + ; moderate intensity:2 + ; strong intensity:3 + ) and positive proportion (Score = staining intensity × positive proportion).

### Transwell assays

Boyden chambers (aperture: 8 µM, 24 wells; BD Biosciences, NJ, USA) were applied for cell migration and invasion analysis. Cells were isolated, rinsed with PBS, and resuspended in a serum-free medium. For invasion assay, chambers were placed in 24 wells plate containing medium with 20% FBS and coated with 60 μl matrigel, cell suspension (1×10^5^ cells/200 µl) was added to each upper chamber and cultured for 48 h. After fixed in methanol and stained with Giemsa (Solarbio, Shanghai, China), the invasive cells on lower surface of filter were photograph taken under microscope and counted with Image Analysis System. For cell migration assay, chambers were used free from Matrigel, 5 × 10^4^ / 200 μl cells were added and cultured for 24 h, the other steps were identical to that of cell invasion assay.

### Cell viability assay

Cell viabilites were measured by Cell Counting Kit-8 (CCK8) assay (Promega, Madison, Wisconsin, USA) or recorded by Real-time Cell Analysis xCELLigence (RTCA) system (Applied Biosystems, Foster City, CA, USA). Briefly, 1×10^3^ cells were planted in 96-well plates or E-plate16 (ACEA Biosciences Inc, CA, USA). The proliferation status of cells could be reflected by absorbance at 450 nm (CCK8 assay) or cell index (RTCA) at respective time points. Three technical repetitions were performed.

### 5-ethynyl-2’-deoxyuridine (EdU) staining

EdU assays were performed with Cell-Light EdU Apollo 567 in vitro imaging kit (RiboBio, Guangzhou, China). Briefly, 5 × 10^3^ BC cells were inoculated into a 15 mm confocal dish (Biosharp life sciences, Beijing, China) and incubated overnight. After being cultured overnight, cells were incubated with 100 μl 50 μM EdU buffer for 2 h, cells were then fixed and permeated with 4% paraformaldehyde and 0.5% Triton X-100, respectively. After washing twice with PBS, cells were incubated with 100 μl of 1X Apollo^®^ staining reaction solution at room temperature in a dark environment for 30 min. Finally, the dishes were sealed with parafilm containing DAPI following the permeabilization process, and the pictures were photographed using a Zeiss ZFM-700 fluorescence microscope (Heidenheim, Germany).

### RNA-pull down and mass spectrometry (MS) analysis

The biotin-labeled probes of circSLC38A1 and control were designed by Sangon Bio (Shanghai, China). In brief, 1×10^7^ cells were washed in ice-cold PBS, and lysed in 500 μl lysis buffer. Then, 3 μg biotinylated DNA oligo probes targeting the junction sequence of circSLC38A1 or control probe were used for incubating with streptavidin magnetic beads at 20-35 °C with rotation for 2 h. Following a thorough washing with PBS, the proteins pulled down in the beads were eluted by incubated cell lysis at 4 °C overnight. SDS-PAGE was performed to separate and quantify the expression of target proteins. Gels were stained with Coomassie blue, cut at 100 kDa, and then subjected to MS analysis. The circSLC38A1 probe was as follows: GACACTCAATAAATATAGAAAAAGTAG GATTCC.

### RNA immunoprecipitation (RIP)

RIP experiments were conducted using a Magna RIP Kit (series number: 17700, Millipore) referring to the manufacturer’s instructions. T24 cells were lysed in complete RNA immunoprecipitation (RIP) lysis buffer, and the cell extract was incubated with magnetic beads conjugated with anti-ILF3, anti-IGF2BP2 (Cat No: 11601-1-AP, proteintech) and anti-IGF2BP3 (Cat No: 14642-1-AP, proteintech) with rotation at 4 °C overnight. After, proteinase K was used to eliminate the proteins bound to the beads. MeRIP experiments were performed with a Magna MeRIP™ m6A Kit (Cat. 17-10499) (Millipore) according to the manufacturer’s instructions. Co-precipitated RNA was detected by qRT-PCR.

### Cycloheximide (CHX) treatment

BC cells transfected with respective constructs were treated by 100 μg/ml for the indicated time, then, the total protein of cells was extracted. The expression of ILF3 protein was quantified via electrophoresis, and thereby the effect of circSLC38A1 on the half-life of ILF3 was evaluated.

### In vitro ubiquitination assay

Briefly, HEK293T cells were co-transfected with several indicated constructs. Cells were collected and lysed after MG132 treatment for 6 h. Then, the protein extracted from cells was incubated with anti-Flag M2 Magnetic Beads (Lot#SLCF4223) (Sigma) at 4 °C. After incubation overnight, the magnetic beads were collected and washed 5 times in PBST for 5 min. Then the protein pulled down by magnetic beads was denatured by incubating with 30 μl 2× SDS sample at 95 °C for 10 min buffer and resolved by SDS-PAGE for immunoblotting.

### Cleavage under targets and tagmentation (CUT&Tag) sequencing

CUT&Tag assays were conducted as previously described [27]. Briefly, T24 cells were pre-treated with ConA-beads. Then we use digitonin to increase the permeability of the T24 cell membrane. Added anti-ILF3 binds to the target chromatin protein between nucleosomes in the genome, and the excess was washed away. After DNA purification, genomic fragments with adapters at both ends were enriched by PCR, sequencing was performed (Jiayin Biomedical Technology, Shanghai, China), and target genes were analyzed. The GEO Accession number is GSE186225.

### Statistics

All experiments were performed in triplicate. Data conformed to a normal distribution as assumed and were presented as mean ± SD. Comparison between two groups was analyzed using the Student’s *t*-test and comparisons among multiple groups were analyzed using a one-way analysis. The chi-square test was employed to verify the association between circSLC38A1 and clinic pathological characteristics. The variance was mild within each group of data and similar between the groups that were being statistically compared. Statistical analyses were performed using GraphPad Prism (v8.0.1, GraphPad Software Inc., San Diego, CA, USA).

## Results

### circRNA expression profiles in BC

**Fig. 1:**
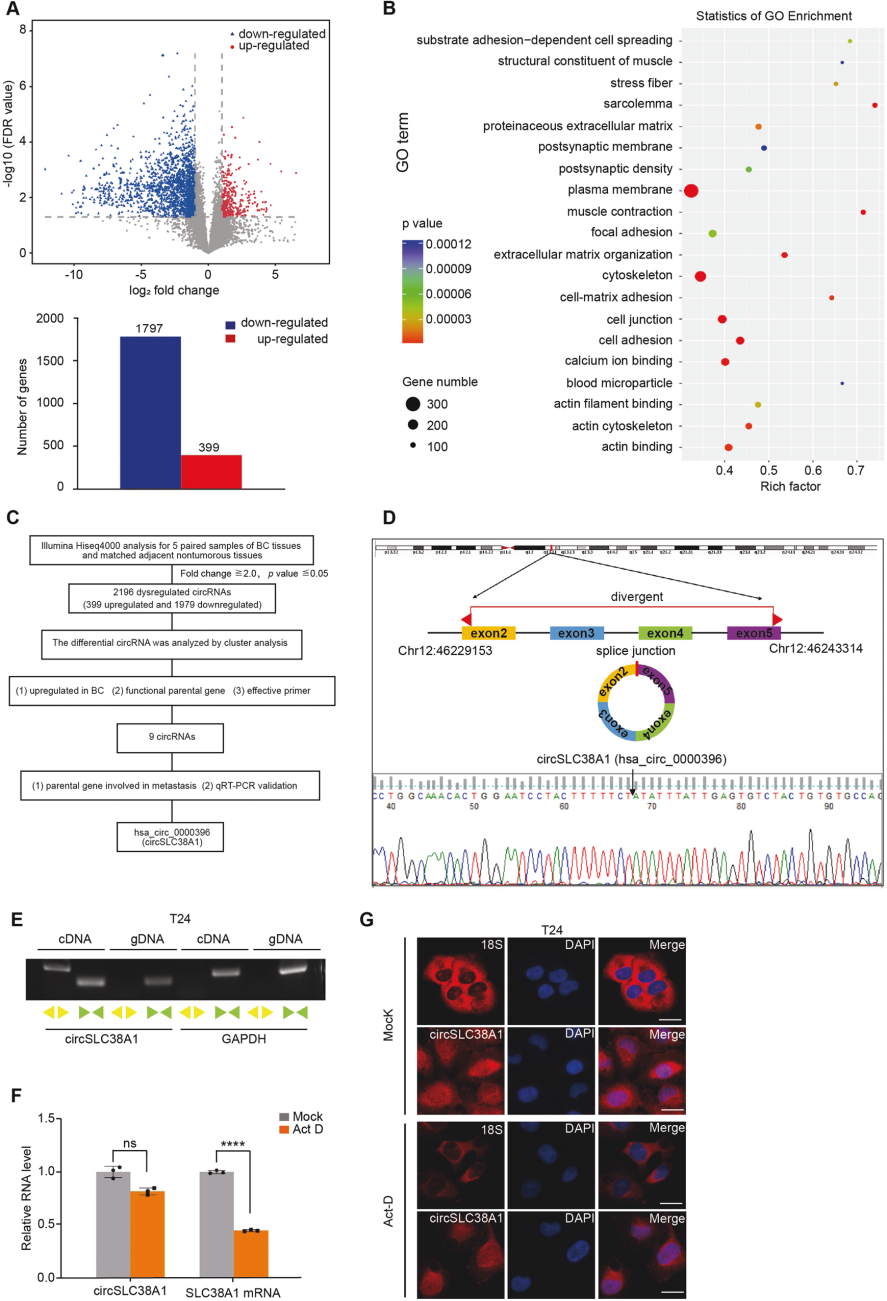
circRNA expression profiles in BC. **A** Top: volcano plot of the differentially expressed circRNAs in five pairs of BC tissues and corresponding adjacent nontumorous tissues by RNA-seq analysis. X-axis: log2 ratio of circRNA expression levels between normal and tumor tissues. Y-axis: the false discovery rate value (−log10 transformed) of circRNAs. Bottom: a total of 2196 dysregulated circRNAs were identified in BC tissues, of which 399 circRNAs were upregulated and 1797 circRNAs were downregulated (*P* < 0.05 and fold change >2.0). **B** GO analysis for the 2196 dysregulated circRNAs. **C** The flowchart delineates the steps for identifying circRNAs in BC**. D** Illustration of the annotated genomic region of SLC38A1, the putative different mRNA splicing forms (linear splicing and ‘head-to-tail’ splicing), circSLC38A1 is back-spliced by exon 2–5 of SLC38A1. Sanger sequencing following PCR conducted using the indicated divergent flanking primers showed the ‘head-to-tail’ splicing of circSLC38A1 in T24 cells. **E** CircSLC38A1 and GAPDH were amplified from cDNA or gDNA from T24 cells with divergent and convergent primers, respectively. **F** Stability of circSLC38A1 and linear SLC38A1 in T24 cells with or without 2 μg/mL Act D treatment for 24 h compared by qRT-PCR. ****p < 0.0001, error bars represent three independent experiments. **G** FISH with junction-specific probe of circSLC38A1 and 18 S probe was used to detect the localization of circSLC38A1; scale bar: 25 μm.

High-thought sequencing analysis of 5 paired BC and adjacent normal tissues was carried out to explored the expression profiles of circRNA. Principal component analysis (PCA) analysis showed a good grouping condition (Fig. S1a). 2196 circRNAs differentially expressed in BC tissues were screened, among which 399 were upregulated, and 1797 were downregulated (Fig. 1A). The parental genes of differentially expressed circRNAs were subjected to GO analysis. The results showed that these dysregulated genes participated in a wide range of important cellular activities, such as cell migration and invasion (Fig. 1B). To validate the potential functional circRNAs, we verified targeted circRNAs with a multi-step pipeline (Fig. 1C). Detailly, we firstly screened with our specialized rules: 1) upregulated in BC and fold change > 2.0; 2) parental gene was reported to participate in cancer progression, and 3) could be effectively detected with specific primers. On this basis, a series of circRNAs were obtained (Table S6). Among those candidate genes, hsa_circ_0000396, which derived from exon 2 to 5 of solute carrier family 38 member 1 (SLC38A1) gene with a length of 522 nt, we designated as circSLC38A1, attracted our attention since SLC38A1 was essential for cell migration and tumor metastasis [28, 29]. We next assessed the sequence and loop structure of circSLC38A1, as the sanger sequencing data shown, the sequence amplified by divergent primers, which specific targeted the back-spliced junction of circSLC38A1, is consistent with circBase database annotation (Fig. 1D). In addition, the reverse-transcribed RNA (cDNA) could be amplified by using divergent primers while genomic DNA (gDNA) could was not detected (Fig. 1E), which rules out possibilities that the observed head-to-tail splicing was produced by trans-splicing genomic rearrangements or even PCR artifacts. Analysis for the stability of circSLC38A1 and its linear form in T24 cells treated with Actinomycin D, an inhibitor of transcription, revealed that circSLC38A1 was more stable than linear SLC38A1 (Fig. 1F). Also, circSLC38A1 was observed to resist digestion by RNase R (Fig. S1b). Finally, we evaluated its subcellular distribution by performing the FISH assay with a junction-specific probe. As shown, circSLC38A1 was located in both the nucleus and cytoplasm of T24 cells (Fig. 1G).

### circSLC38A1 is upregulated in BC

**Fig. 2:**
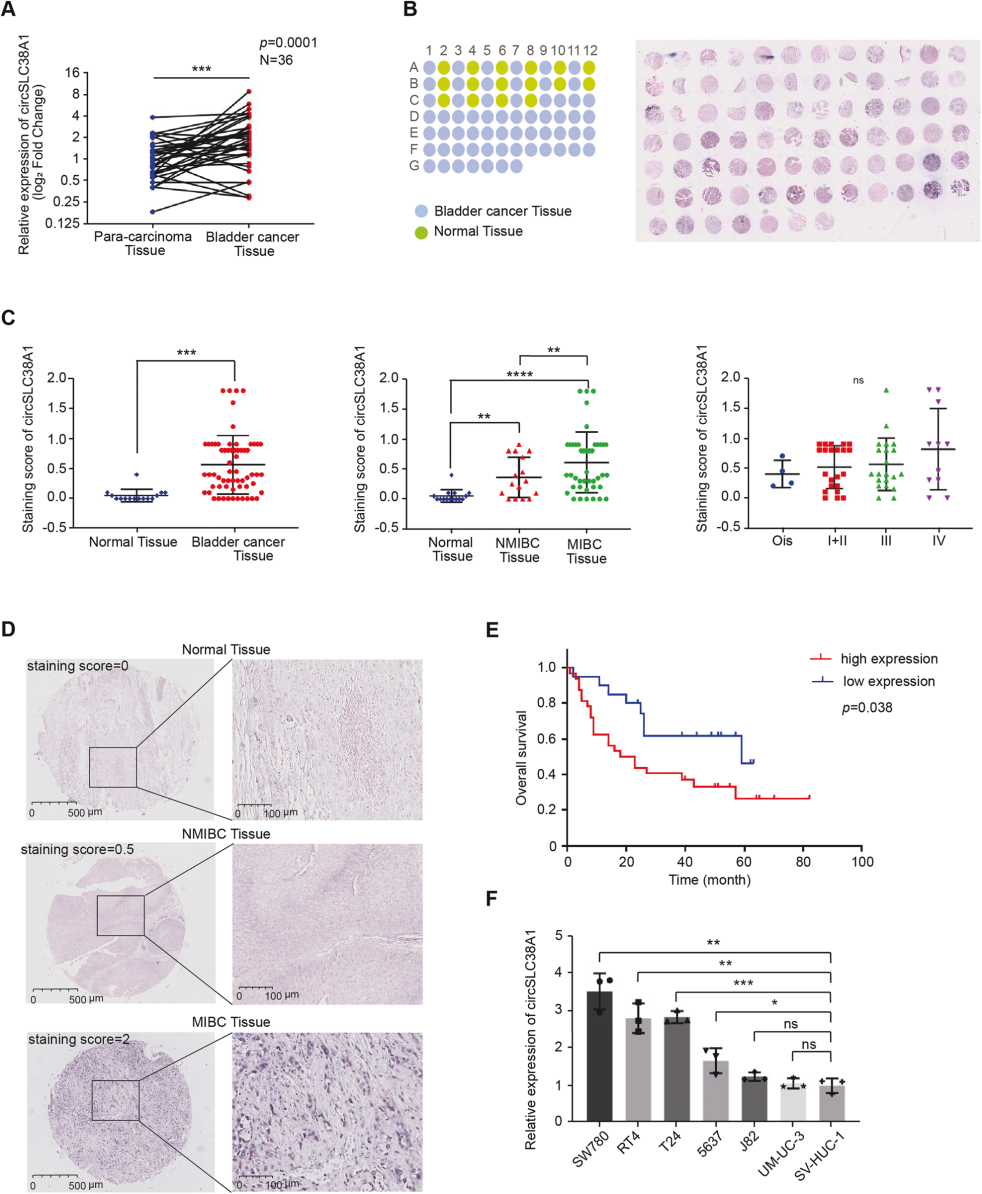
circSLC38A1 is upregulated in BC. **A** Expression levels of circSLC38A1 in 36 pairs of BC and adjacent normal tissues were detected by the divergent primers (*P* = 0.0001). **B** The expression level of circSLC38A1 was measured by RNA ISH staining tissue microarrays (*n* = 79). **C** The difference in staining score between normal tissues and BC tissues (left), the difference in staining score between NMIBC tissues and MIBC tissues (middle), and the correlation of circSCL38A1 expression with tumor node metastasis classification (TNM) of bladder cancer (left). The staining score = staining intensity score * staining positive rate. (staining intensity score 0 is defined as negative, staining intensity score 1+ is defined as weak expression, staining intensity score 2+ is defined as moderate expression, and staining intensity score 3+ is defined as a strong expression). **D** The representative staining images were shown. **E** Kaplan–Meier analysis of the correlation between circSLC38A1 expression and overall survival. Patients with high levels of circSLC38A1 had significantly shorter overall survival (*P* = 0.038). The *P* value was determined by a Log-rank test. **F** Relative expression of circSLC38A1 in BC cell lines and human normal urothelial cell line measured by qRT-PCR. Data are presented as means ± standard deviation from three independent experiments. *p < 0.05, **p < 0.01, ***p < 0.001, ****p < 0.0001.

To verify the RNA-seq data and determine whether circSLC38A1 participates in BC progression, we measured its expression in 36 pairs of BC and adjacent normal tissues via qRT-PCR. Consistent with the RNA-seq analysis, circSLC38A1 was significantly upregulated in BC tissues in contrast to that in adjacent paired normal samples (Fig. 2A). Meanwhile, the expression of other circRNAs was not significantly altered. Furthermore, circSLC38A1 expression levels in the TMA tissues from Cohort II (containing 63 BC tissues and 16 non-tumor tissues) were determined by RNA-IHC analysis (Fig. 2B). As shown in Fig. 2C, circSLC38A1 expression was elevated in BC tissues when compared to normal ones; moreover, dramatically higher tumoral circSLC38A1 levels were also verified in 47 muscle-invasive bladder cancer (MIBC) tissues in comparison with 16 non-muscle-invasive bladder cancer (NMIBC) tissues; and the the expression of circSLC38A1 increased with increasing TNM stage, although there is no significant statistical difference in the results (Fig. 2C). Three representative histologic images are shown in Fig. 2D. In addition, higher tumoral circSLC38A1 levels (staining score >0.3) were correlated with shorter overall survival (OS) (Fig. 2E). Consistently, circSLC38A1 was upregulated in some BC cell lines (SW780, RT4, T24 and 5637) compared to human normal urothelial cell line, SV-HUC-1 (Fig. 2F). The above results suggested that circSLC38A1 was upregulated and might play a pivotal role in BC progression.

### CircSLC38A1 promoted aggressive phenotypes of BC

**Fig. 3:**
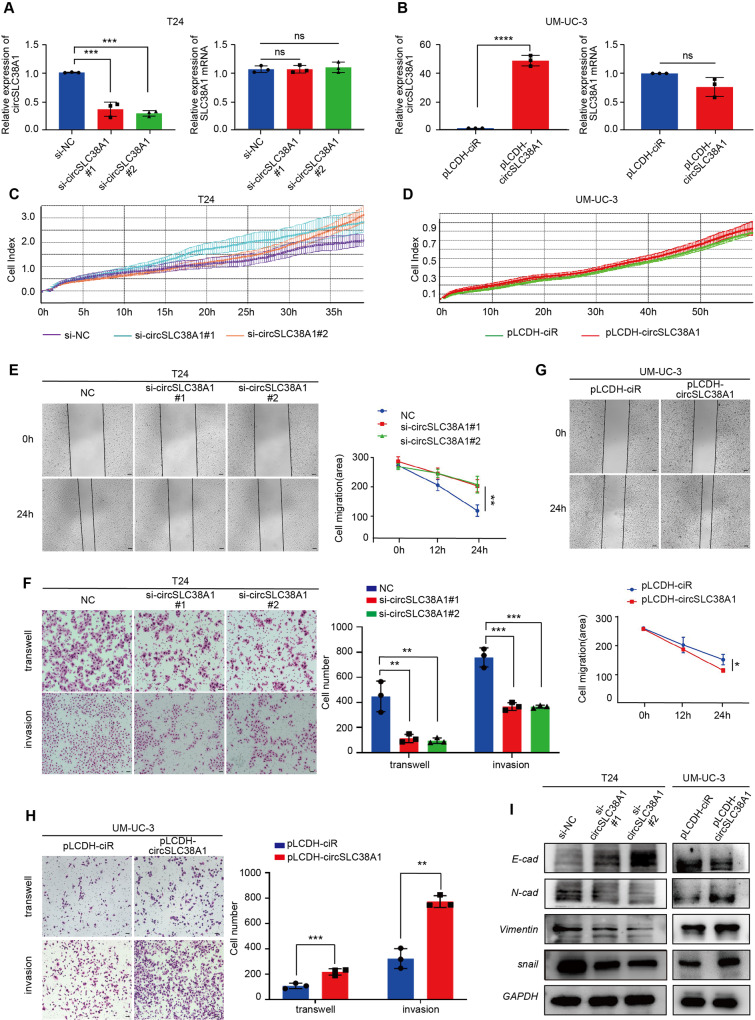
circSLC38A1 promotes migration and invasion capacities of BC cells in vitro. **A** Relative expression levels of circSLC38A1 and SLC38A1 mRNA in T24 cells treated with circSLC38A1 siRNA or corresponding negative control (si-NC). **B** Relative expression levels of circSLC38A1 and SLC38A1 mRNA in UM-UC-3 cells after transduction with circSLC38A1 overexpression plasmid or vector plasmid. **C** The cell viability of T24 cell lines transfected with si-circSLC38A1 or si-NC were recorded by RTCA system. **D** The cell viability of UM-UC-3 cell lines transfected with circSLC38A1 overexpression plasmid or vector plasmid were recorded by RTCA system. **E**, **F** Wound healing assay (**E**), transwell migration, and matrigel invasion assay (**F**) showing decreased cell invasion in circSLC38A1 knockdown T24 cells. Scale bar, 100 µm (**E**), 25 µm (**F**). **G**, **H** Wound healing assay (**G**), transwell migration and matrigel invasion assay (**H**) showing that increased cell invasion in circSLC38A1 overexpression UM-UC-3 cells. Scale bar, 100 µm (**G**), 25 µm (**H**). **I** The expression levels of E-cadherin, N-cadherin, Vimentin, and snail in BC cells with knockdown or overexpression of circSLC38A1 were detected by western blot. Data are presented as means ± standard deviation from three independent experiments. *P < 0.05, **P < 0.01, ***P < 0.001.

To further explore the biological significance of circSLC38A1 in BC progression, gain- and loss-of-function studies were performed. Short interfering RNAs and overexpression plasmids were designed according to the sequence and back-splicing junction of circSLC38A1 (Fig. S1c). T24 and 5637 cells were selected for the following loss-of-function assays due to their relatively higher endogenous expression of circSLC38A1, whereas UM-UC-3 and J82 cells with relatively lower circSLC38A1 expression were selected for gain-of-function assays (SW780 and RT4 cells were not selected due to the features of growing with clumps and had a significantly prolonged doubling time). The silencing and overexpressing effects were confirmed by performing the FISH analysis and qRT-PCR assay (Fig. 3A, B and Fig. S1d–f). Meanwhile, the SLC38A1 mRNA level was not altered upon transfection of respective oligonucleotides, confirming the specificity of circSLC38A1 vectors (Fig. 3A, B, Fig. S1e and f). RTCA, CCK8 and EdU assays showed that dysregulated circSLC38A1 in T24 and UM-UC-3 cells had little effect on cell proliferation (Fig. 3C, D and Fig. S1g–j). However, migration and invasion assays revealed that silencing circSLC38A1 could markedly suppressed the mobility of T24 and 5637 cells (Fig. 3E, F and Fig. S2b). Consistently, overexpression of circSLC38A1 in T24, UM-UC-3, and J82 cells distinctly increased cell migration and invasion (Fig. 3G, H and Fig. S2a, c). Western blot and qRT-PCR revealed that dysregulated circSLC38A1 significantly affected the phenotype of epithelial-mesenchymal transition (EMT), such as the negatively regulated epithelial marker, E-cadherin; and the positively regulated mesenchymal marker Vimentin, N-cadherin, and Snail (Fig. 3I and Fig. S2d–k).

**Fig. 4:**
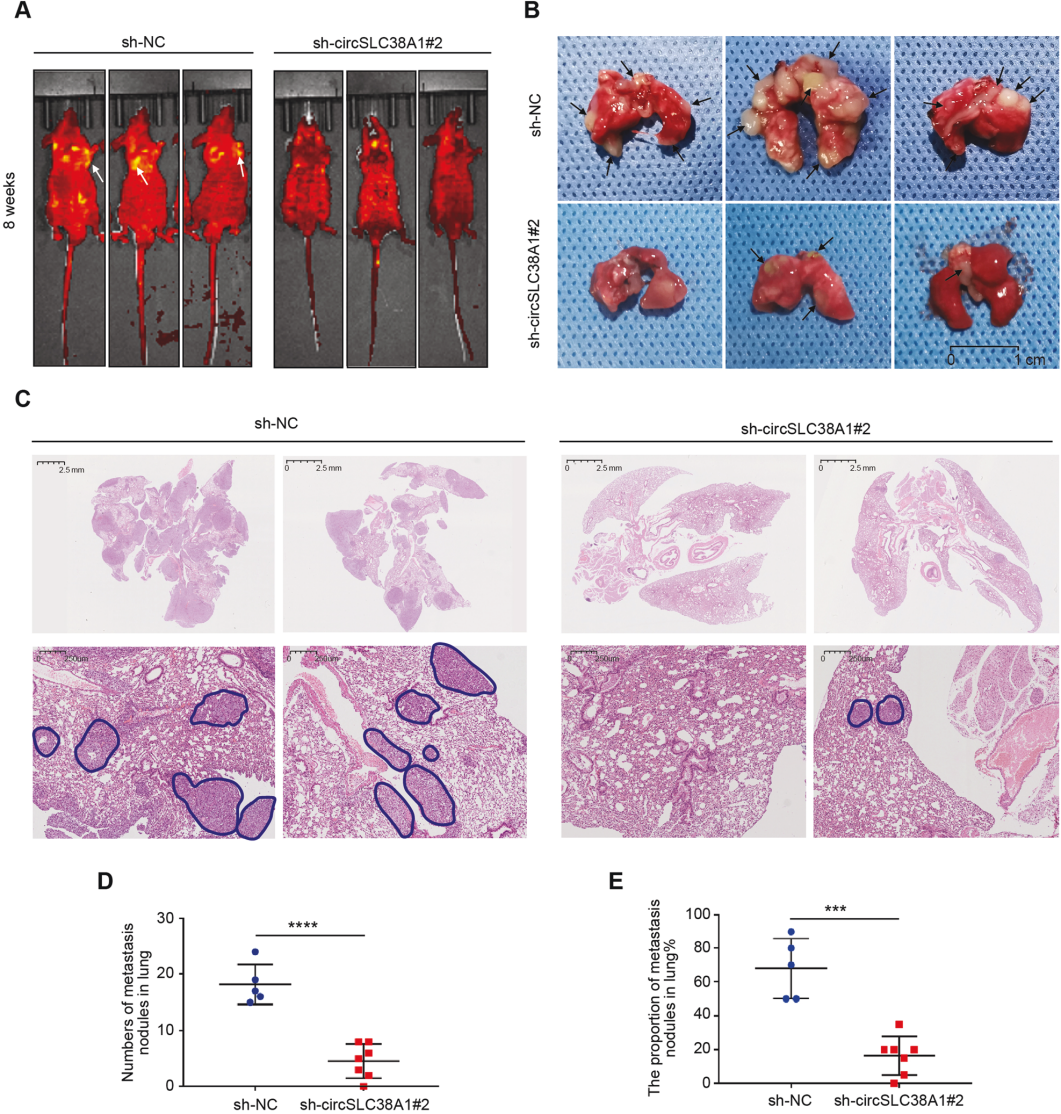
circSLC38A1 promotes metastasis of BC in vivo. **A** Representative fluorescent images for each experimental group at 8 weeks. **B** Representative images about tumor metastasis formed in the lungs of mice through vein tail injection of circSLC38A1-NC or circSLC38A1- knockdown BC cells. Metastatic nodules formed in the lungs of mice (Black Arrow). **C** Left, representative H&E staining of lung metastatic lesion through vein tail injection of circSLC38A1-NC BC cells. Multiple tumor metastases can be seen in the lung tissue. Right, representative H&E staining of lung metastatic lesion through vein tail injection of circSLC38A1-knockdown BC cells. The area circled with blue lines showed the tumor nests, among which the tumor cells exhibited solid growth mode. **D** The number of metastatic nodules formed in the lungs of mice for each group. **E** The proportion of metastatic nodules in the lungs of mice for each group. Data represent mean ± SD, and the *P* values were determined by a two-tailed unpaired Student’s *t* test, ***p < 0.001, ****p < 0.0001.

Given the importance of circSLC38A1 based on in vitro observations, we performed in vivo experiments to validate its essential role in BC. Metastatic mice model was generated by tail vein injection of sh-circSLC38A1 T24 cells; we found that circSLC38A1 expression was significantly lower in tumors generated from cells infected with sh-circSLC38A1 than in control vectors (Fig. S3a). Tumor metastasis in the lung was significantly inhibited after the silence of circSLC38A1 in BC cells (Fig. 4A, B). H&E staining further confirmed the malignancy of the sh-circSLC38A1#2 group was impaired (Fig. 4C). Quantitative data on the number of metastatic nodules also supported our conclusion (Fig. 4D, E). To further eliminate the potential cytotoxicity of injected vectors and their influence on cell proliferation, we detected cell proliferation via RTCA assay and IHC staining of Ki-67. As shown, there is no difference in cell viability between WT cells and NC vector cells (Fig. S3b). Meanwhile, the silence of circSLC38A1 showed no effect on Ki-67 expression (Fig. S3c), which excluded the potential in vivo effect of circSLC38A1 on xenograft growth. Collectively, circSLC38A1 possessed oncogenic-like functions in promoting invasion and metastasis in BC.

### CircSLC38A1 interacted with ILF3 protein

**Fig. 5:**
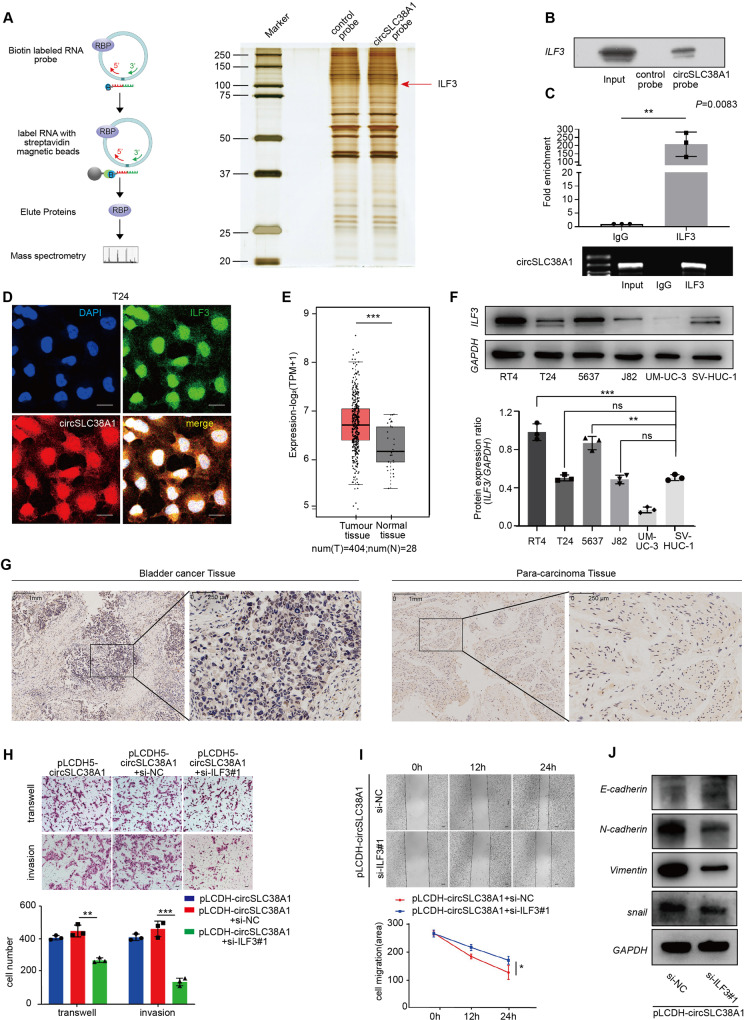
CircSLC38A1 interacted with ILF3 protein. **A** Left, schematic diagram showing the process of circSLC38A1-pull down. The biotin-labeled probes were used to pull down interacting proteins in T24 cells, the pull-down proteins were identified by MS. Right, identification of the circSLC38A1-protein complex pulled down by circSLC38A1 probe with protein extracts from T24 cells. The arrows indicate the additional band presented in circSLC38A1-protein complex. **B** Immunoblot analysis of ILF3 after pulldown assay showing its specific association with circSLC38A1. **C** RIP assays showing the association of ILF3 with circSLC38A1 in T24 cells. Top, fold enrichment representing RNA levels associated with ILF3 relative to IgG, IgG antibody served as a control. Bottom, agarose gel electrophoresis for products of RIP assay. **D** Co-localization of circSLC38A1 and ILF3 visualized by coupling circRNA FISH assay with ILF3 Immunofluorescence staining; scale bar: 25 μm. **E** ILF3 is up-regulated in BC based on data available from TCGA database. **F** Relative expression of ILF3 in BC cell lines and human normal urothelial cell line detected by western blot. **G** Expression levels of ILF3 in BC and adjacent normal tissues were detected by immunohistochemical staining. **H**, **I** Interference with ILF3 can effectively reverse the migratory and invasive potential of bladder cancer cells induced by circSLC38A1. **J** The expression levels of E-cadherin, Vimentin, ILF3, and snail between pLCDH-circSLC38A1+si-NC and pLCDH-circSLC38A1 + siILF3^#^1 group were detected by western blot. Data represent mean ± SD from three independent experiments; *P < 0.05, **P < 0.01, ***P < 0.001.

Having observed a pro-metastatic role of circSLC38A1 in BC, we next sought to determine the molecular mechanism behind this role. Previous studies demonstrated that circRNAs may interact with RBPs to form an RNA-protein complex and thereby regulate the activity of target genes at the transcriptional level [19, 30]. To investigate this possibility and identify putative circSLC38A1-interacted proteins, The RNA pull-down assay followed by MS analysis was conducted in the T24 cell. The RNA-related proteins were determined using SDS-PAGE and silver staining (Fig. 5A). By using MS and comparing the results with those from the control group, proteins specifically associated with circSLC38A1 were identified (Table S7). ILF3 was chosen due to the highest expression in the circSLC38A1 probe compared with the control probe sample. Western blot analysis with anti-ILF3 antibody indicated the existence of ILF3 within the pull-down samples of circSLC38A1-probe (Fig. 5B). To further confirm the interaction between circSLC38A1 and ILF3, we performed a RIP assay. Results showed that endogenous ILF3 was enriched at circSLC38A1 in T24 cells (Fig. 5C). Then we coupled circRNA in situ hybridization assay with an immunofluorescence staining for ILF3 protein and verified that circSLC38A1 could co-localize with ILF3 protein in the nucleus (Fig. 5D). These complementary experiments consistently indicated that endogenous circSLC38A1 could interact with ILF3 mainly in the nucleus, where ILF3 mostly resides (Fig. S4a).

By analyzing the datasets from GEPIA 2 (http://gepia2.cancer-pku.cn/#index), we found that ILF3 is up-regulated in various cancers, including BC (Fig. 5E and Fig. S4b). The upregulation of ILF3 transcript was further confirmed in 34 paired BC and adjacent tissues (Fig. S4c). It is worth noting that a positive correlation between the expression level of ILF3 and circSLC38A1 (Fig. S4d). In addition, higher ILF3 levels seemed to correlate with poor disease-free survival in BC patients (Fig. S4e). We also confirmed that ILF3 protein was upregulated in several BC cell lines and tumor tissue by western blot and immunohistochemical staining, respectively (Fig. 5F, G). Based on these findings, we aimed to investigate the possibility that the engagement of ILF3-mediated circSLC38A1-induced metastasis. To prove this hypothesis, we designed three independent siRNAs used for silencing ILF3. Compared with the scramble control, siILF3#1 and siILF3#2 could effectively knock down ILF3, either at the transcription level or protein level (Fig. S4f); thus, siILF3#1 and siILF3#2 were selected for follow-up experiments. As shown, the silence of ILF3 significantly decreased the invasive ability of BC cells (Fig. S4g). ILF3 inhibition strikingly reversed the oncogenic properties caused by upregulated circSLC38A1, including the migratory, invasive capacities and EMT phenotype (Fig. 5H–J, Fig. S4j), we also verified the biological function of ILF3 in another BC cell line, overexpression of ILF3 could effectively restore the migratory ability that reduced due to circSLC38A1 knockdown (Fig. S4k). Taken together, these lines of evidence suggest circSLC38A1 could directly interact with ILF3, thereby exerting functional roles in BC.

### circSLC38A1 increased ILF3 expression via inhibition of protein degradation

**Fig. 6:**
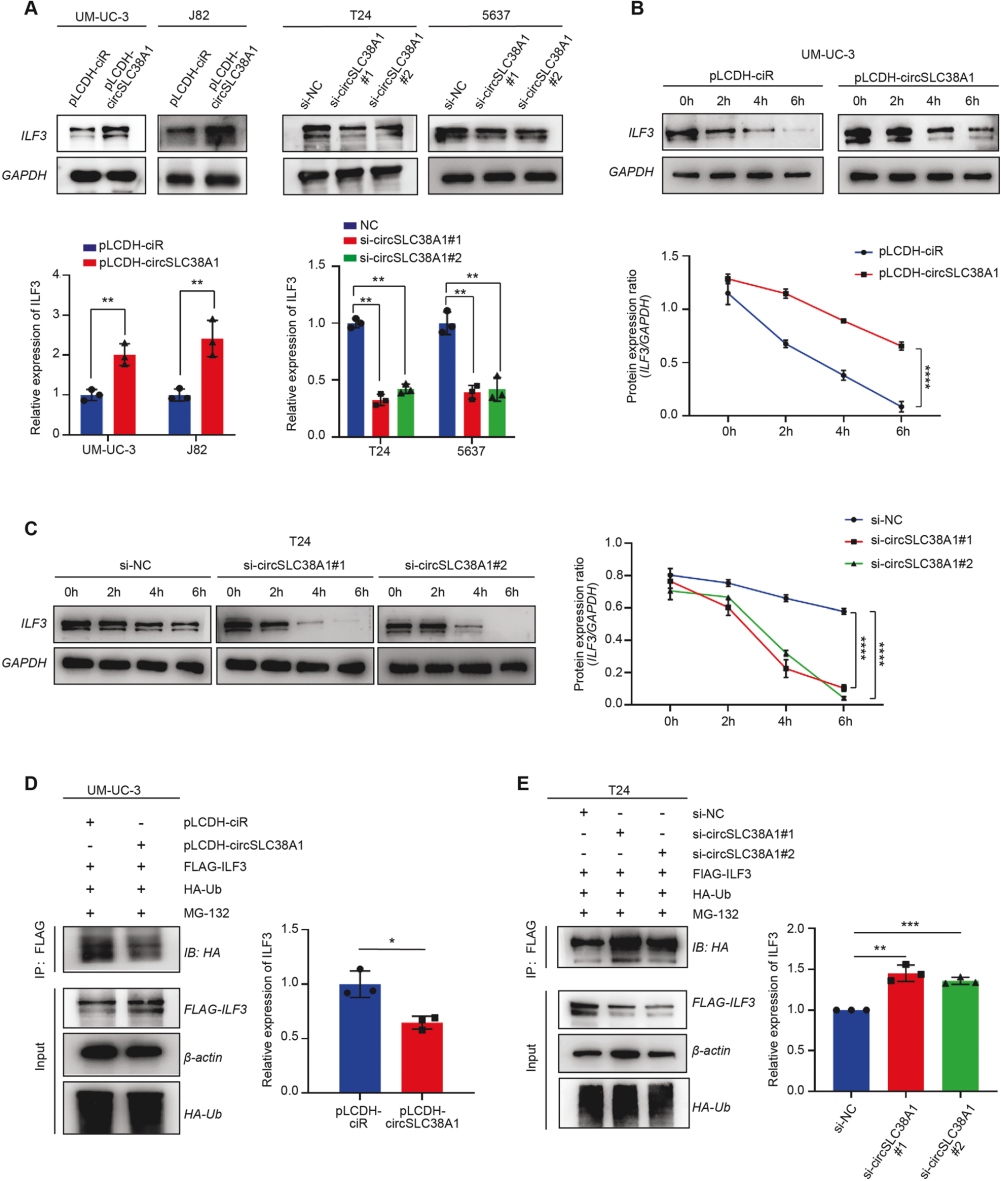
circSLC38A1 increased ILF3 expression via inhibition of protein degradation. **A** Expression levels of ILF3 in BC cells with circSLC38A1 deficient or circSLC38A1 overexpression. **B** Protein level of ILF3 in UM-UC-3 cells over-expressed circSLC38A1, followed by 100 µg/ml CHX treatment for the indicated time points. **C** Protein level of ILF3 in circSLC38A1 knockdown T24 cells, followed by 100 µg/ml CHX treatment for the indicated time points. **D**, **E** Immunoblot analysis of ILF3 in polyubiquitination assays of 293 T cells transfected with the indicated constructs and treated with 20 µM MG132 for 6 h. Data represent mean ± SD from three independent experiments; *P < 0.05, **P < 0.01, ***P < 0.001, ****P < 0.0001.

Once it was clear that circSLC38A1 functions, at least in part, through the interaction with ILF3, we sought to investigate the mechanisms underlying this regulation mode. First, qRT-PCR assay confirmed that ILF3 mRNA level was not altered regardless of the presence of circSLC38A1 or not (Fig. S4h, i), which ruled out the possibility that circSLC38A1 could transcriptionally modulate ILF3 expression. Intriguingly, the knockdown of circSLC38A1 reduced ILF3 protein level in both T24 and 5637 cells, whereas forced expression of circSLC38A1 in UM-UC-3 and J82 cells increased the level of ILF3 protein (Fig. 6A). This leads us to test whether circSLC38A1 modulates ILF3 expression through impairing protein degradation. With the treatment of BC cells with CHX, we evaluated ILF3 levels at different time points. As shown in Fig. 6B, C, the half-life of ILF3 was prolonged when circSLC38A1 was overexpressed and decreased when circSLC38A1 was silenced, suggesting that circSLC38A1 mediated ILF3 upregulation through suppressing protein degradation. Then we conducted a ubiquitination IP assay using 293T cells, which are more effective in generating multiple-transfection phenotype cells. Our results showed that circSLC38A1 overexpression could increase ILF3 steady-state expression, retarded ILF3 poly-ubiquitination, and decelerated ILF3 protein turnover, while knockdown of circSLC38A1 resulted in the opposite effects (Fig. 6D, E). These data suggest that circSLC38A1 upregulated ILF3 expression via inhibition of protein degradation.

### CircSLC38A1 recruits ILF3 to initiate TGF-β2 expression

**Fig. 7:**
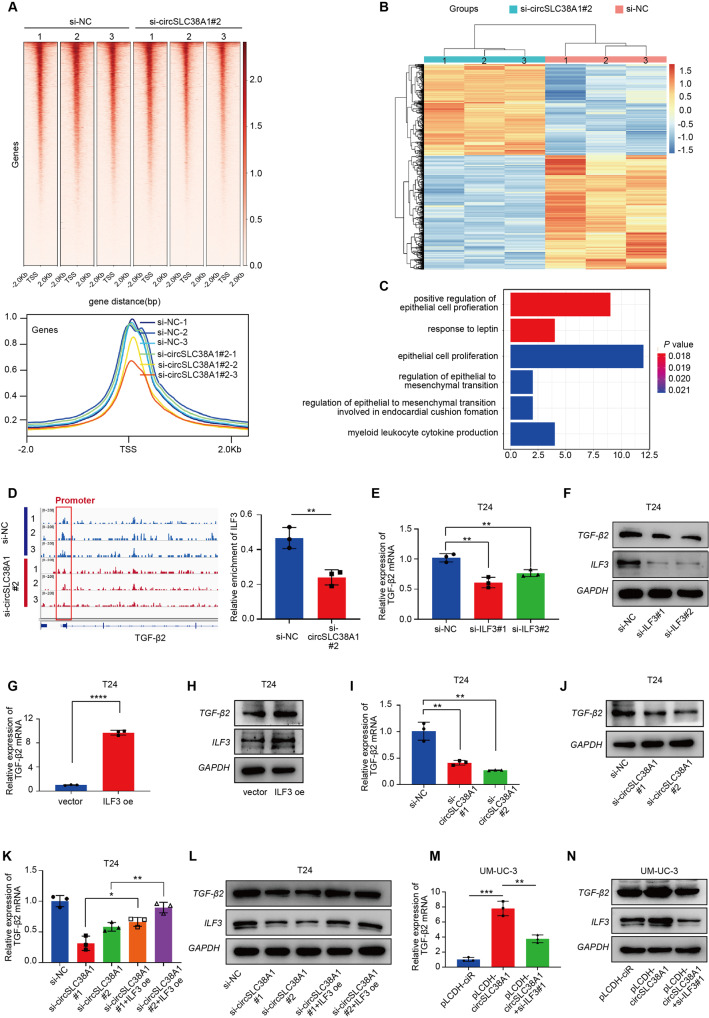
CircSLC38A1 recruits ILF3 to initiate TGF-β2 expression. **A** Top: Heatmap of CUT&Tag-seq peaks associated with the ILF3 in T24 cell lines transfected with si-circSLC38A1#2 or si-NC, signals are displayed from -2.0 kb to +2.0 kb surrounding the TSS. Bottom: the average intensity curves for ILF3 signals at TSSs in a region comprising ±2 Kb in T24 cell lines transfected with si-circSLC38A1 or si-NC. **B** Heatmap of the differentially expressed RNAs in si-circSLC38A1 and si-NC cells by RNA-seq analysis. Red, upregulated RNAs; blue, downregulated RNAs. **C** Functional enrichment analysis of overlapped downregulated genes that were uniquely aligned to the genome using the online DAVID software. The results show that these genes are significantly associated with functional modules, such as regulation of epithelial cell proliferation and regulation of epithelial to mesenchymal transition. **D** Right: the IGV shows the CUT&Tag signals of ILF3 at the TGF-β2 gene loci. Left: quantitative diagram of the degree of enrichment of ILF3 signal on the TGF-β2 promoter. **E**, **F** Relative expression levels of TGF-β2 in T24 cells treated with ILF3 siRNA or corresponding negative control were measured by qRT-PCR (**E**) or western blot (**F**). **G**, **H** Relative expression levels of TGF-β2 in T24 cells transfected with vector control or ILF3 overexpression plasmid were measured by qRT-PCR (**G**) or western blot (**H**). **I**, **J** Relative expression levels of TGF-β2 in T24 cells treated with circSLC38A1 siRNA or corresponding negative control were measured by qRT-PCR (**I**) or western blot (**J**). **K**, **L** The expression of TGF-β2 in T24 cells with circSLC38A1 deficiency could be effectively reversed by overexpression of ILF3. **M**, **N** The expression of TGF-β2 that was upregulated with circSLC38A1 overexpression could be reduced by knocking down the expression of ILF3. Data represent mean ± SD from three independent experiments; *P < 0.05, **P < 0.01, ***P < 0.001, ****P < 0.0001.

Based on the co-localization of circSLC38A1 and ILF3 in the nucleus, we hypothesized that ILF3 might function as a transcription factor by binding to the promoter area of downstream target genes. To prove the above hypothesis, we performed CUT&Tag-seq using anti-ILF3 antibody after transfection of si-circSLC38A1. The heat map showed that peaks mainly enriched in TSS regions, and compared to the control group, the signal enriched by ILF3 protein in the si-circSLC38A1 groups were notably weaker (Fig. 7A). The sequences around CUT&Tag-seq peaks were highly conserved, and the peaks were frequent in promoters, introns, and intergenic regions (Fig. S5a, b). We also analyzed the differentially expressed genes after circSLC38A1 deletion using RNA sequencing. The heatmap and principal component analysis (PCA) disclosed that the si-circSLC38A1 group and control could be differentiated by the RNA expression profile (Fig. 7B and Fig. S5c). A total of 912 dysregulated RNAs were identified between the circSLC38A1-deficient group and control group, among which 405 were upregulated, and 507 RNAs were downregulated (*P* < 0.05 and fold change >2.0) (Fig. S5d). By integrated analysis of CUT&Tag-seq and RNA-seq data, we identified 172 downregulated genes in the si-circSLC38A1 group potentially regulated by ILF3 (Fig. S5e). Furthermore, sixteen genes can be enriched by GO analysis and transforming growth factor beta-2 (TGF-β2), and NOG was chosen since they were enriched to epithelial to mesenchymal transition (EMT) process (Table S8, Fig. 7C). TGF-β2 was a well-reported oncogene involved in tumor metastasis [15], and we found that the expression level of TGF-β2 increased with tumor TNM staging progress through TCGA database analysis (Fig. S5f). The IGV map clearly showed that the proximal promoter region of TGF-β2 was occupied by ILF3 protein, and importantly, this occupancy was reduced by silencing circSLC38A1 (Fig. 7D and Fig. S5g). By detecting the expression level of TGF-β2 and NOG2 upon transfection of si-ILF3 or ILF3 overexpression plasmid, we found that NOG2 level was not influenced (Fig. S5h). At the same time, ILF3 regulated TGF-β2 at both transcript and protein levels (Fig. 7E–H, Fig. S5i, j). Consistently, TGF-β2 was also downregulated by knocking down of circSLC38A1 (Fig. 7I, J and Fig. S5k); however, this effect was partially reversed by the overexpression of ILF3 (Fig. 7K, L and Fig. S5l). We also carried out rescue experiments in UM-UC-3 cells, as shown in Fig. 7M, N and Fig. S5m, the level of TGF-β2 protein and mRNA which up-regulated by circSLC38A1 overexpression was reduced by knocking down ILF3. Our integrated analysis revealed that circSLC38A1-ILF3 complex might induce BC progression via transcriptionally regulation of TGF-β2 expression.

### m6A modification may related to the upregulation of circSLC38A1 in BC cells

**Fig. 8:**
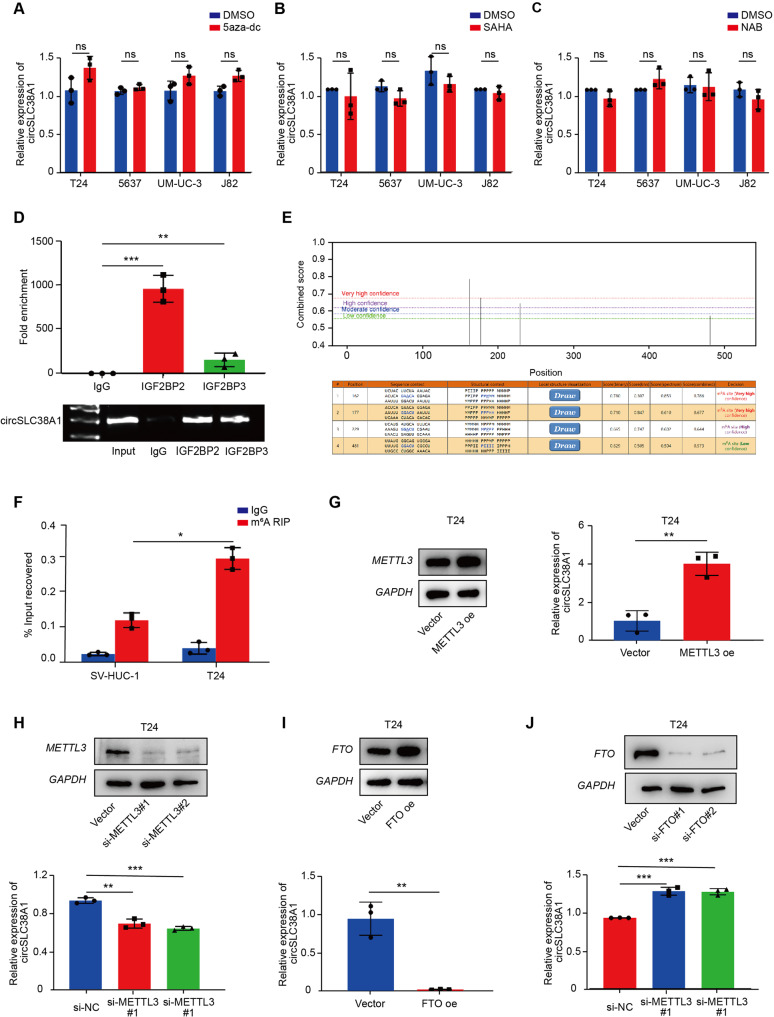
m6A modification is involved in the upregulation of circSLC38A1 in BC cells. **A** 4 BC cells were treated with or without 5 µM 5-aza-dC for 7 days, and circSLC38A1 expression was measured by qRT-PCR. **B** 4 BC cells were treated with or without 2 µM SAHA for 24 h, and circSLC38A1 expression was measured by qRT-PCR. **C** 4 BC cells were treated with or without 2 µM NAB for 24 h, and circSLC38A1 expression was measured. **D** RIP assays shows the association between IGF2BP2 or IGF2BP3 with circSLC38A1 in T24 cells. Top, fold enrichment representing RNA levels associated with IGF2BP2 or IGF2BP3 relative to IgG, IgG antibody served as a control. Bottom, agarose gel electrophoresis for products of RIP assay. **E** Three m6A modification sites on circSLC38A1 with high or very high confidence were identified by using online software tool SRAMP. **F** m6A RIP qRT-PCR analysis of circSLC38A1 in T24 and SV-HUC-1 cells. **G** The expression levels of circSLC38A1 in T24 cells transfected with vector control or METTL3 overexpression plasmid were measured by qRT-PCR. **H** The expression levels of circSLC38A1 in T24 cells transfected with si-NC or METTL3 siRNAs were measured by qRT-PCR. **I** The expression levels of circSLC38A1 in T24 cells transfected with vector control or FTO overexpression plasmid were measured by qRT-PCR. **J** The expression levels of circSLC38A1 in T24 cells transfected with si-NC or FTO siRNAs were measured by qRT-PCR. Data represent mean ± SD from three independent experiments; *P < 0.05, **P < 0.01, ***P < 0.001.

It is widely reported that epigenetic regulations are critical for gene expression [31, 32]. To investigate whether circSLC38A1 expression levels was regulated by epigenetic mechanisms, we treated BC cells with a DNA methyltransferase inhibitor 5-aza-dc, and HDAC inhibitors, SAHA and NAB, respectively. Intriguing, circSLC38A1 expression was affected neither by 5-aza-dc treatment (Fig. 8A) nor SAHA or NAB treatment (Fig. 8B, C), indicating that DNA methylation and histone acetylation were not associated with the upregulation of circSLC38A1 in BC.

Previous studies revealed that the m6A-guided splicing events positively correlated with elevated levels of circRNAs [33], suggesting that enriched m6A levels may increase circRNA biogenesis. To find the potential role of m6A modification in circSLC38A1 expression, we predicted m6A-related proteins using the online software, *CircInteractome*. IF4A3, IGF2BP2 and IGF2BP3 were predicted to bind circSLC38A1 at the junction site (Fig. S6a). RIP assay proved that IGF2BP2 and IGF2BP3 could directly interact with circSLC38A1 (Fig. 8D). Taking a step further, we used the online software tool *SRAMP (*http://www.cuilab.cn/sramp/*)* to screen potential m6A modification sites on the circSLC38A1 sequence. Three m6A modification sites with high or very high confidence were identified (Fig. 8E and S6b). By designing primers that target m6A modified sites 1 and 2, we performed m6A RNA-immunoprecipitation (MeRIP) to detected whether the circSLC38A1 was modified by m6A. The results showed that the level of circSLC38A1 enrichment by m6A antibody in T24 cells was significantly higher than that in bladder epithelial SV-HUC-1 cells (Fig. 8F).

To further identify the m6A “writer” and “eraser” proteins involved in regulating circSLC38A1, we analyzed the TCGA database. As shown, the “writer” protein METTL3 and “eraser” protein FTO were differentially expressed in BC (Fig. S6c, d). We then evaluate the METTL3 and FTO expression in tissue samples and verify their correlation with circSCL38A1 expression. Consistent with TCGA data analysis, METTL3 was highly expressed in tumor tissues (Fig. S6e). And there was a positive correlation between the expression level of METTL3 and circSLC38A1 (Fig. S6f). By generating respective vectors, we found that enhanced METTL3 increased while knockdown of METTL3 suppressed circSLC38A1 expression in BC cells (Fig. 8G, H). Consistently, upregulated FTO caused the decrease of circSLC38A1 expression while silencing FTO induced upregulation of circSLC38A1 (Fig. 8I, J). Taken together, these data indicated that the m6A modifications might contribute to the upregulation of circSLC38A1 in BC.

### Exosomal circSLC38A1 may serve as a promising serum biomarker for BC patients

Circular RNAs are enriched and stable in exosomes [34], indicating exosomal circRNA could serve as a promising biomarker for cancer diagnosis. To investigate whether circSLC38A1 can be packaged into exosomes, we extracted exosomes from the serum of Cohort III (65 BC patients and 64 non-cancerous donors). The detailed information of BC patients from cohort III was presented in Table S2. Through TEM observation, the extracted exosomes showed typical membrane vesicles with diameters between 80 and 150 nm (Fig. S7a). NTA showed the exosomes’ size distribution, and the diameter was verified at ~109 nm (Fig. S7b). Expression of the well-accepted exosome marker proteins, CD9, CD63, and Tumor Susceptibility Gene 101 (TSG101), were identified by western blot (Fig. S7c). We then detected circSLC38A1 expression in exosomes and found that circSLC38A1 level was detectable and abundant in serum exosomes derived from BC patients compared to non-cancerous donors subject (Fig. S7d). Moreover, dramatically higher exosomal circSLC38A1 levels were identified in MIBC patients compared to NMIBC patients (Fig. S7e). ROC curve analysis showed the diagnostic value of exosomal circSLC38A1 in discriminating BC from a healthy population was remarkably high (AUC = 0.878) (Fig. S7f) and an AUC value of 0.729 in distinguishing MIBC from NMIBC (Fig. S7g). These data indicate that serum exosomal circSLC38A1 may be used as a promising blood test indicator for BC patients.

## Discussion

circRNAs are widespread and stable transcripts in eukaryotic cell organisms and are highly correlated with cancer status and prognosis in a tissue- and developmental-stage-specific manner [35, 36]. Bladder cancer is characterized as an invasive disease, and invasive muscle status has been considered an independent prognostic factor. However, the clinically effective indicator is limited, and great efforts were devoted to finding novel, useful predictive markers. Therefore, we proposed to verify a useful circRNA marker and investigated its potential role in BC metastasis and prognosis [37]. In this study, by performing deep RNA sequencing and clinical tissue specimen verification, we identified a novel circRNA, circSLC38A1, was elevated and associated with poor prognosis in BC. We further revealed the potential role of the tumor promoter of circSLC38A1 by conducting in vitro and in vivo assays.

There are currently few reports describing the role of circRNAs interacting with protein, forming specific circRNPs complex, and subsequently influencing the expression or function associated proteins [19, 20]. However, whether circRNAs play essential roles in BC metastasis is unclear. Our data identified ILF3 as the binding partner for circSLC38A1 and potentiated their regulatory activities post-translational. Moreover, circSLC38A1 consequently blocked ILF3 ubiquitination and degradation and thus maintained the stability and expression of ILF3. Notably, we show that ILF3 inhibition strikingly rescued the oncogenic properties caused by upregulated circSLC38A1, which means circSLC38A1 functions at least in part through the interactions with ILF3. To this end, this is a frontier study revealing the role of circRNAs via binding with TF proteins, thereby activating the transcription of BC metastasis-related oncogenes. Meanwhile, the binding domain of ILF3 will be clarified by analyzing the spatial structure, and the mechanism by which circSLC38A1 inhibits ILF3 ubiquitination will be explored in our future studies.

ILF3 was tightly correlated with pathogenesis, progression, drug resistance, and prognosis in various cancers [23–26, 38]. As an RNA-binding protein, ILF3 participates in a variety of cellular functions through binding to different cellular RNAs, Interestingly, increasing studies showed that ILF3 could bind to RNA and bind to DNA in mammalian cells. ILF3 has been shown to have isoforms with different functions in both transcription activation and repression depending on the genome location of the binding site [21]. For example, ILF3 could bind to IL2 or VEGF promoters and promote its transcription [39, 40]. Using CUT&Tag sequencing and RNA sequencing, we identified TGF-β2 as the downstream targets of ILF3 in BC cells. The mechanism by which TGFβ/SMADs signaling regulates tumorigenesis and cancer progression through EMT has been well studied [41]. Here, we proved that TGF-β2 could be regulated by circSLC38A1-ILF3 complex in BC, which complemented the upstream regulatory mechanism of TGF-β2.

The N6-methyladenosine (m6A) modification is a dynamic and reversible process and implicates all stages of the RNA synthesis and maturation, including RNA processing, splicing, and translation, etc. [32], and thus takes part in the regulation and functions of RNAs, including circRNAs [42, 43]. The dysregulations of circRNAs have long been investigated from multiple aspects; recent finding has demonstrated that m6A-guided splicing events could increase circRNA biogenesis [33]. Using online software, we found that circSLC38A1 may interact with m6A readers, such as IF4A3 and IGF2BPs. In addition, the m6A modification sites of circSLC38A1 were also predicted. METTL3-modulated m6A is the most prevalent RNA epigenetic regulation in eukaryotic cells and contributes to multiple pathological processes. In addition, the study has revealed that METLL3 could exhibit distinct patterns of m6A modifications in promoting the formation of m6A-circRNAs, with suggestions that the regulation of m6A-mediated circRNA biogenesis was broadly [43]. After validating the involvement of METTL3 in BC progression, we further provided evidence that circSLC38A1 was specifically targeted and regulated by METTL3-mediated m6A modification, which uncovered the mechanism of how circSLC38A1 was upregulated in BC.

Non-invasive biomarkers or liquid biopsies can revolutionize cancer patient management as repeated sampling allows real-time monitoring of disease progression, which were cornerstones of personalized medicine [44]. Exosomes are nanoscale extracellular vesicles produced by the shedding of most types of cells [45]. The special membrane structure enables them and their contents to exist stably in a variety of body fluids, and confers them and their contents inside the potential to serve as biomarkers to diagnose human disease [46], Exosomal circSATB2 may use as a candidate biomarker for clinical distinguishing of non-small cell lung cancer (NSCLC) and metastatic NSCLC [47]. Our study found circSLC38A1 could be packaged into exosomes, and serum exosomal circSLC38A1 expression was higher in BC patients, especially MIBC patients. More importantly, we demonstrated high diagnostic sensitivity and specificity of serum exosomal circSLC38A1 in differentiating MIBC and NMIBC. These data support the potential of serum exosomal circSLC38A1 as a non-invasive biomarker for BC diagnosis.

There are study limitations in this report. First, the detailed underlying binding mode, including the critical area of ILF3 protein by circSLC38A1 and the binding ratio between ILF3 protein and circSLC38A1, was not clarified due to technical issues. We are now focusing on this topic and will investigate it continuously in our subsequent studies. Second, whether there is any role for the immune system (e.g., infiltration of immune cells) in controlling tumor metastasis is a promising direction, which will help reveal the involvement of tumor immunity in circSLC38A1-mediated BC metastasis.

## Conclusions

In summary, our study demonstrated that m6A methylation-caused dysregulated circSLC38A1 could promote metastasis of BC by binding to and stabilizing ILF3, which further initiated TGF-β2 expression. Moreover, circSLC38A1 can be packaged into exosomes, and the exosomal circSLC38A1 could serve as a promising biomarker for the detection of BC (Fig. S7h). Therefore, our study may help better understand the basic regulatory mechanism and clinical significance of circRNAs in BC.

## Supplementary information

Supplementary Figures and Tables

Full and uncropped western blot

Checklist

## Data Availability

The RNA-sequencing data and CUT & Tag sequencing data are uploaded and available at the Gene Expression Omnibus (GEO) under accession GSE190079, GSE186226, and GSE186225.
