# Supplementary material for: Characterization of circSCL38A1 as a novel oncogene in bladder cancer via targeting ILF3/TGF-β2 signaling axis
Source: Cell Death Dis. 2023 Jan 25;14(1):59. doi: 10.1038/s41419-023-05598-2 (PMC9876890; doi:10.1038/s41419-023-05598-2)
Supplement: Supplementary file 1 — Supplementary Figures and Tables [file 41419_2023_5598_MOESM1_ESM.docx]

**Supplementary Figures and Legends**

**
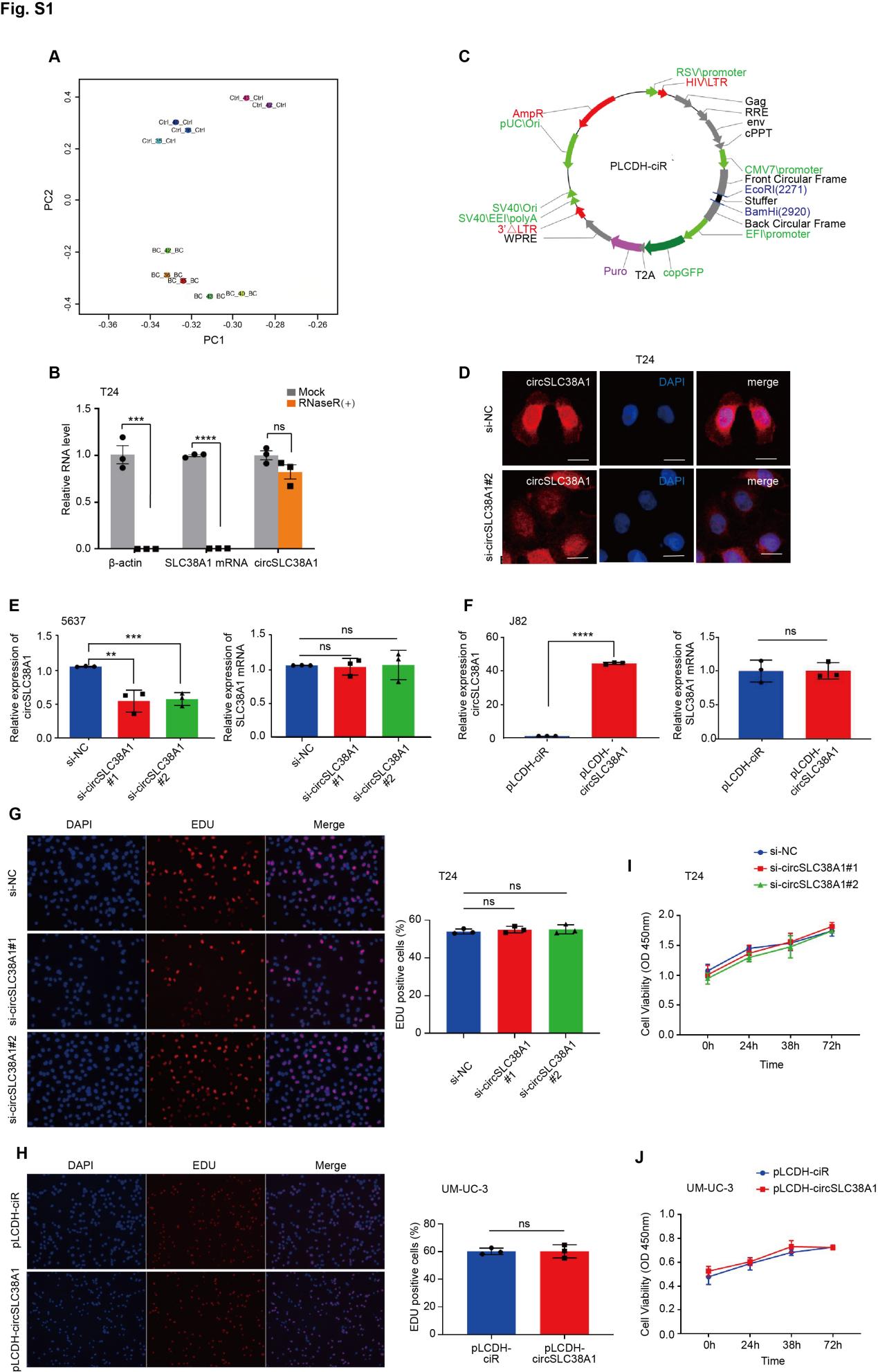
**

**Figure S1. circSLC38A1 contributes less effect on BC cells proliferation.**

**a** The principal component analysis (PCA) disclosed that BC tissues and corresponding adjacent nontumorous tissues could be differentiated by the RNA expression profile. **b** Relative RNA level of circSLC38A1 and linear SLC38A1 treated with RNase R. **c** Schematic view of the pLCDH-ciR vector used to construct the circSLC38A1 overexpression plasmid. **d** FISH with junction-specific probe was used to detect the knockdown effect of circSLC38A1 siRNA; scale bar: 25 μm. **e** Relative expression levels of circSLC38A1 and SLC38A1 mRNA in 5637 cells treated with circSLC38A1 si-circSLC38A1 or si-NC. **f** Relative expression levels of circSLC38A1 and SLC38A1 mRNA in J82 cells after transduction with circSLC38A1 overexpression plasmid or vector plasmid. **g, h** Left, EdU assays to detect proliferation of circSLC38A1-knockdown T24 cells and circSLC38A1 overexpressed UM-UC-3 cells. Right, quantitative analysis of EdU assays. **i, j** The cell viability of BC cell lines was detected by CCK8 in respective cell lines. Data are presented as means ± standard deviation from three independent experiments. **P* < 0.05, ***P* < 0.01, ****P* < 0.001, *****P* < 0.0001.

**
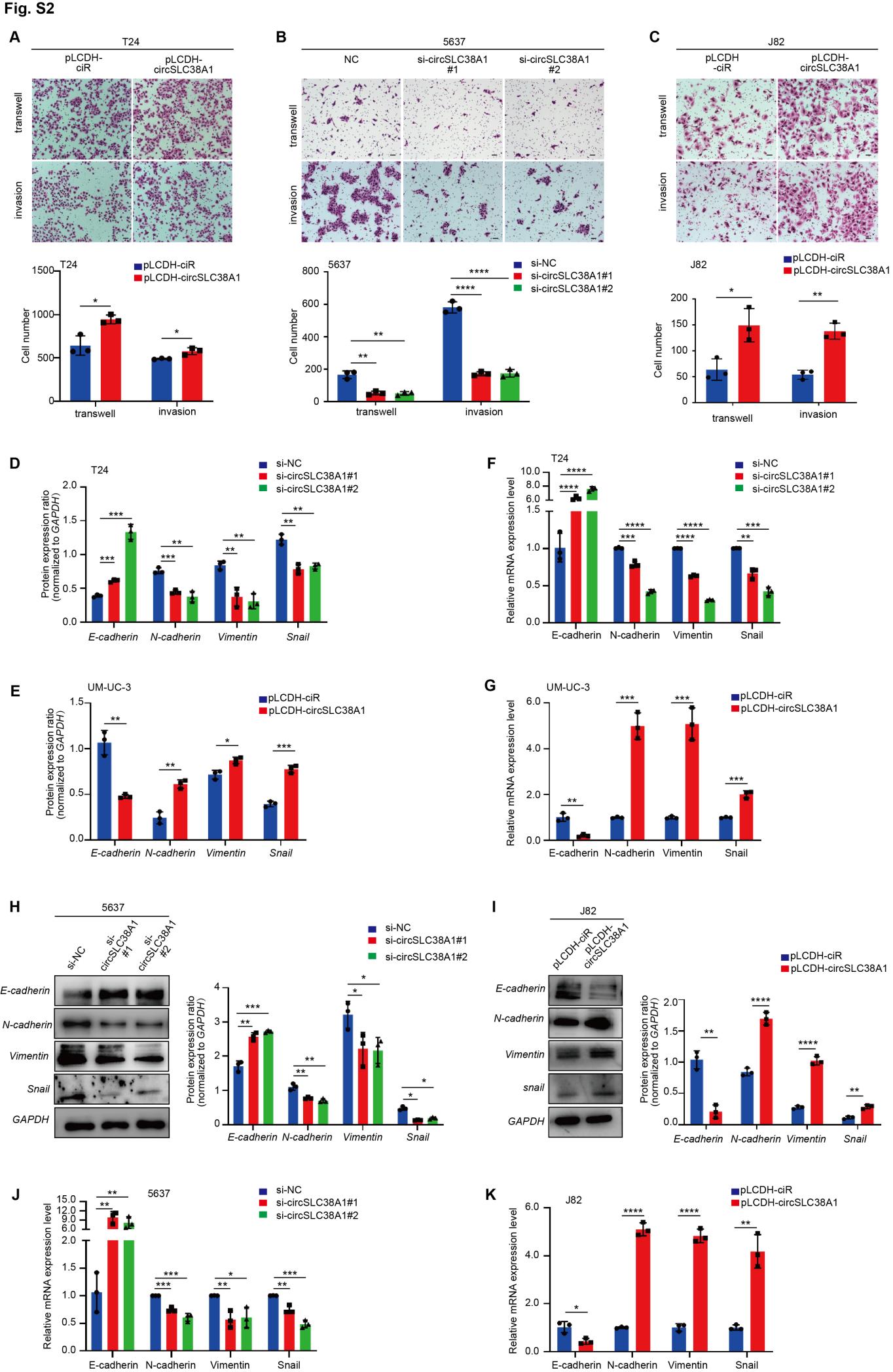
**

**Figure S2. circSLC38A1 promotes migration and invasion capacities of BC cells in vitro.**

**a** Transwell migration and matrigel invasion assays showed that increased cell invasion in circSLC38A1 overexpression T24 cells. Scale bar, 25µm. **b** Transwell migration and matrigel invasion assay showed that decreased cell invasion in circSLC38A1 knockdown 5637 cells. Scale bar, 25µm. **c** Transwell migration and matrigel invasion assay showed that increased cell invasion in circSLC38A1 overexpression J82 cells. Scale bar, 25µm. **d，e** Quantitative diagram of the protein expression levels of E-cadherin, N-cadherin, Vimentin and Snail in circSLC38A1 deficient T24 cells (d), and in circSLC38A1 overexpressed UM-UC-3 cells (e) measured by western blot. **f, g** mRNA levels of E-cadherin, N-cadherin, Vimentin and Snail in circSLC38A1 deficient T24 cells (f), and in circSLC38A1 overexpressed UM-UC-3 cells (g) measured by qRT-PCR. **h, i** The expression levels of E-cadherin, N-cadherin, Vimentin and snail in circSLC38A1 deficient 5637 cells (h), and in circSLC38A1 overexpressed J82 cells (i) were detected by western blot. **j, k** mRNA levels of E-cadherin, N-cadherin, Vimentin and Snail in circSLC38A1 deficient 5637 cells (f), and in circSLC38A1 overexpressed J82 cells (g) measured by qRT-PCR. All experiments were repeated independently three times. Data are presented as means ± SD. **P* < 0.05, ***P* < 0.01, ****P* < 0.001, *****P* < 0.0001.


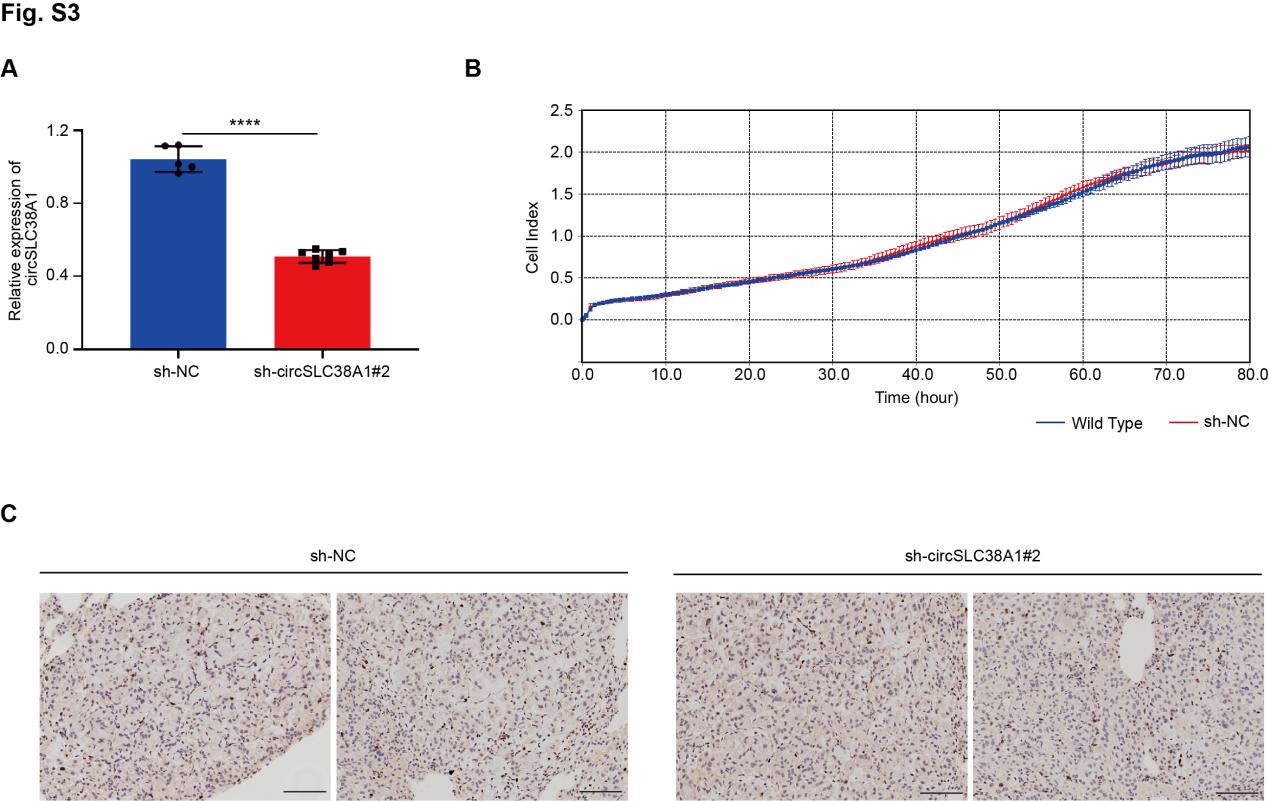


**Figure S3. circSLCA1 contributes less effect on the proliferation of BC in vivo.**

**a** Expression of circSLC38A1 was detected via qRT-PCR in xenograft generated from BC cells infected with sh-NC and sh-circSLC38A1 vectors. **b** The cell viability of T24 cell lines transfected with sh-NC and wild type T24 cells were recorded by RTCA system. **c** Representative ki67 staining of lung metastatic lesion through vein tail injection of circSLC38A1-NC BC cells or circSLC38A1-konckdown BC cells. Data are presented as means ± SD. ***P* < 0.01.

**
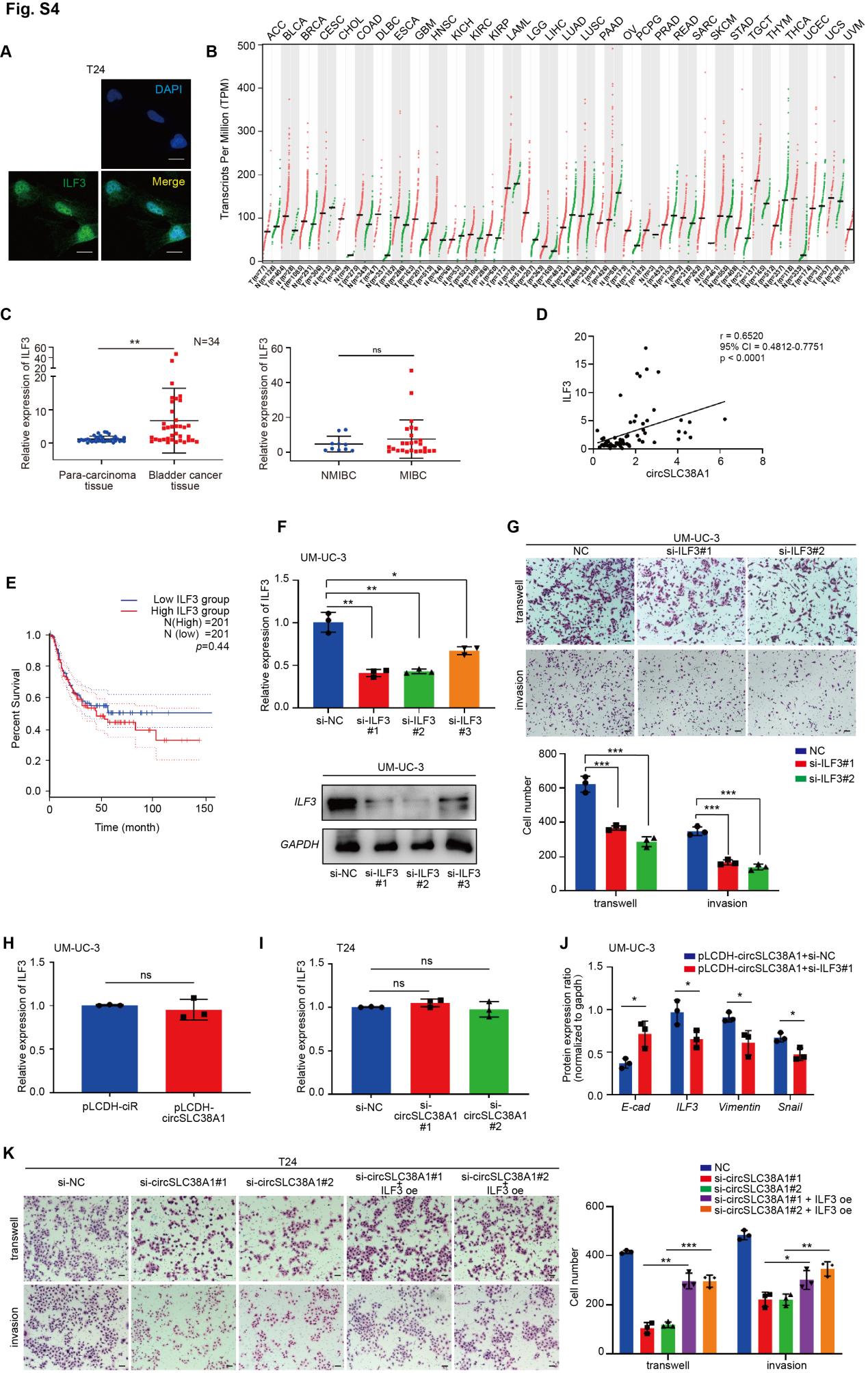

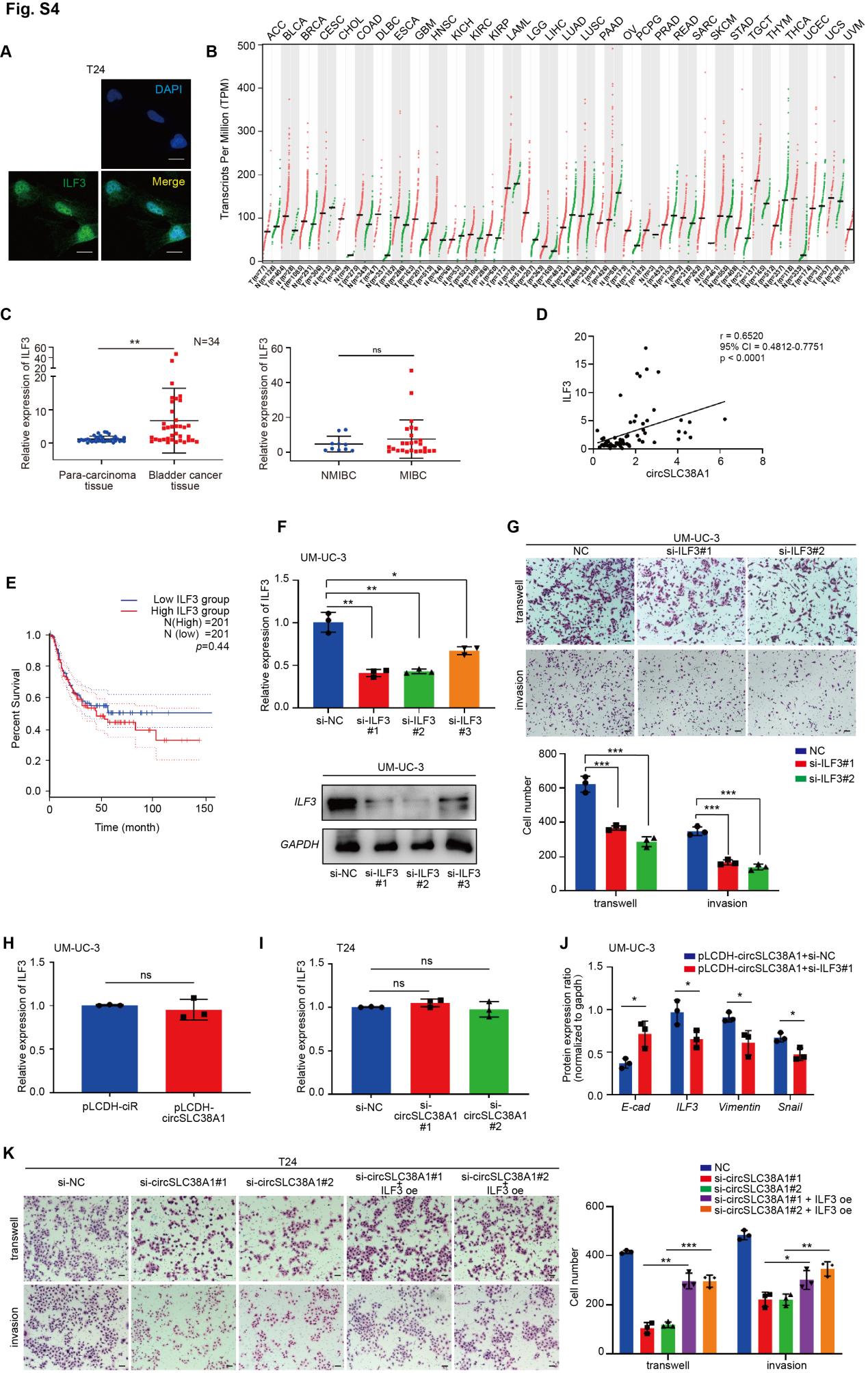
**

**Figure S4. CircSLC38A1 interacted with ILF3 protein.**

**a** Immunofluorescence staining for ILF3 protein showed that ILF3 mostly located at nucleus; scale bar: 25 μm. **b** The gene expression profile showed ILF3 was overexpressed in mostly tumor samples. **c** Left: Expression levels of ILF3 in 36 paired BC and adjacent normal tissues were detected by qRT-PCR (*P* =0.0154). Right: Expression difference of ILF3 between NMIBC tissues and MIBC tissues. **d** The correlation between the expression of ILF3 and circSLC38A1 in samples of Cohort I was analysed using Spearman correlation analysis. **e** Highly expression of ILF3 correlated with shorter disease-free survival time in BC. **f** The efficacy of three siRNAs targeting ILF3 was detected by qRT-PCR and western blot in UM-UC-3 cells. **g** Transwell migration and matrigel invasion assay showed that decreased cell invasion in ILF3 knockdown UM-UC-3 cells. **h, i** The expression levels of ILF3 mRNA with circSLC38A1 overexpression or circSLC38A1 deficient BC cells were detected by qRT-PCR. **j** Quantitative diagram protein of E-cadherin, Vimentin, snail and ILF3 in UM-UC-3 cells transfected with the indicated constructs. **k** Overexpression of ILF3 can effectively restore the migratory and invasive potential of bladder cancer cells impaired by knocking down circSLC38A1. Data represent mean ± S.D. from three independent experiments; **P* < 0.05, ***P* < 0.01, ****P* < 0.001, *****P* < 0.0001.


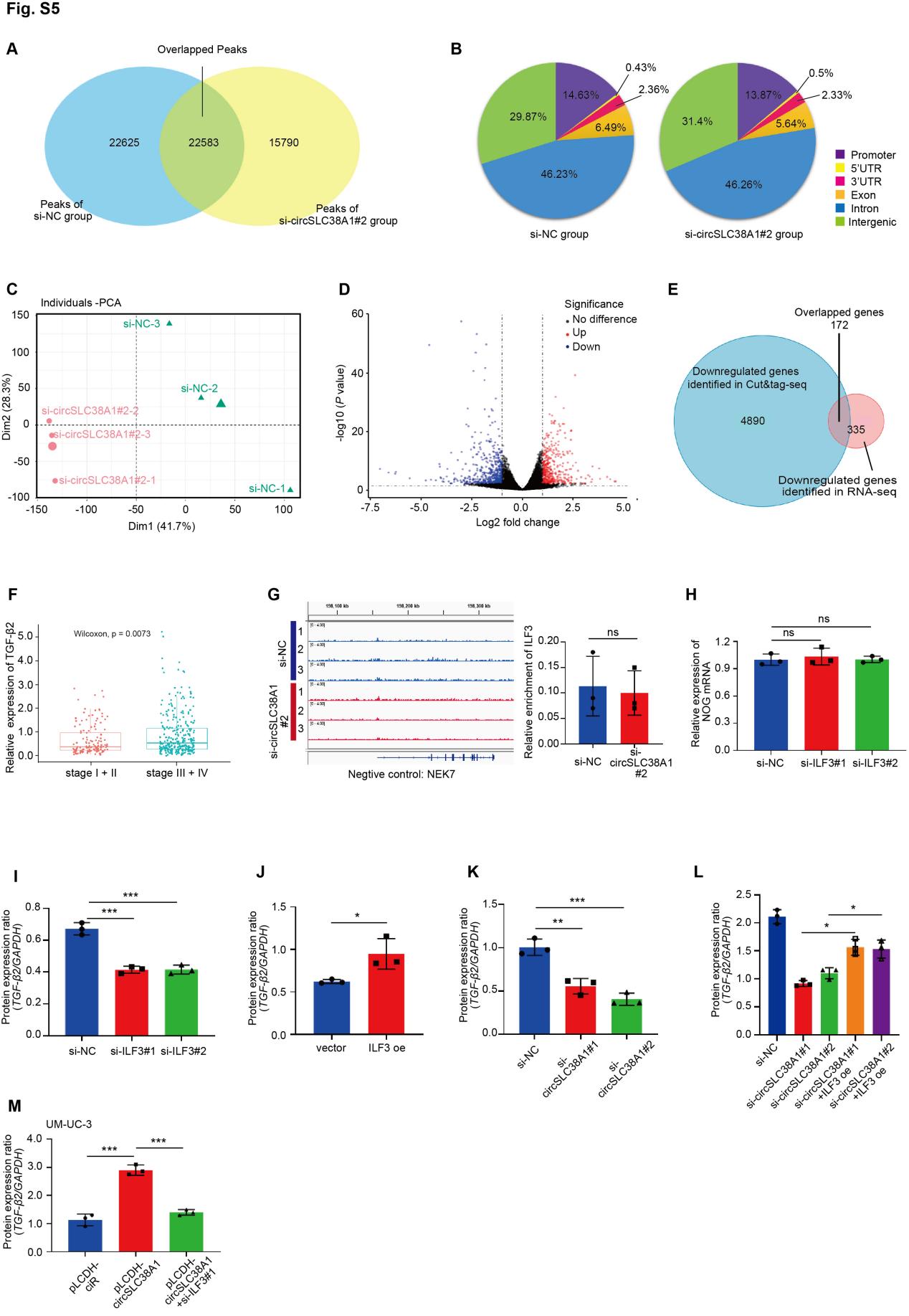


**Figure S5. Determined the downstream targets of circSLC38A1-ILF3 complex by integrating analysis of CUT&Tag-seq and RNA-seq data.**

**a** Venn diagram demonstrating the overlapped peaks enriched by ILF3 protein in the si-circSLC38A1#2 group and the control group. **b** Pie chart showing the distribution of identified peaks in si-circSLC38A1#2 and si-NC group. The sequences around CUT&Tag-seq peaks were highly conserved, and the peaks were frequent in promoters, introns, and intergenic regions. **c** The principal component analysis (PCA) disclosed that si-circSLC38A1#2 group and controls could be differentiated by the RNA expression profile. **d** Volcano plot of RNA expression, a total of 912 dysregulated RNAs were identified between circSLC38A1 deficient group and control group, of which 405 were upregulated and 507 RNAs were downregulated. **e** Venn diagram demonstrating the overlap of downregulated genes between CUT&Tag-seq and RNA-seq. **f** The expression of TGF-β2 increased with tumor TNF staging progress through TCGA database analysis. **g** Right: the IGV showing the CUT&Tag signals of ILF3 at the NEK7 gene loci, NEK7 server as negative control. Left: quantitative diagram of the degree of enrichment of ILF3 signal on the NEK7 promoter. **h** Relative expression levels of NOG in T24 cells treated with ILF3 siRNA or corresponding negative control were measured by qRT-PCR. **i, j** Quantitative diagram of the protein levels of TGF-β2 in T24 cells with ILF3 deficient (**i**) or ILF3 overexpression (**j**). **k** Quantitative diagram of the protein levels of TGF-β2 in T24 cells with circSLC38A1 deficient. **l** Quantitative diagram protein of TGF-β2 in T24 cells transfected with the indicated constructs. **m** Quantitative diagram protein of TGF-β2 in UM-UC-3 cells transfected with the indicated constructs. Data represent mean ± S.D. from three independent experiments; **P* < 0.05, ***P* < 0.01, ****P* < 0.001, *****P* < 0.0001.

**
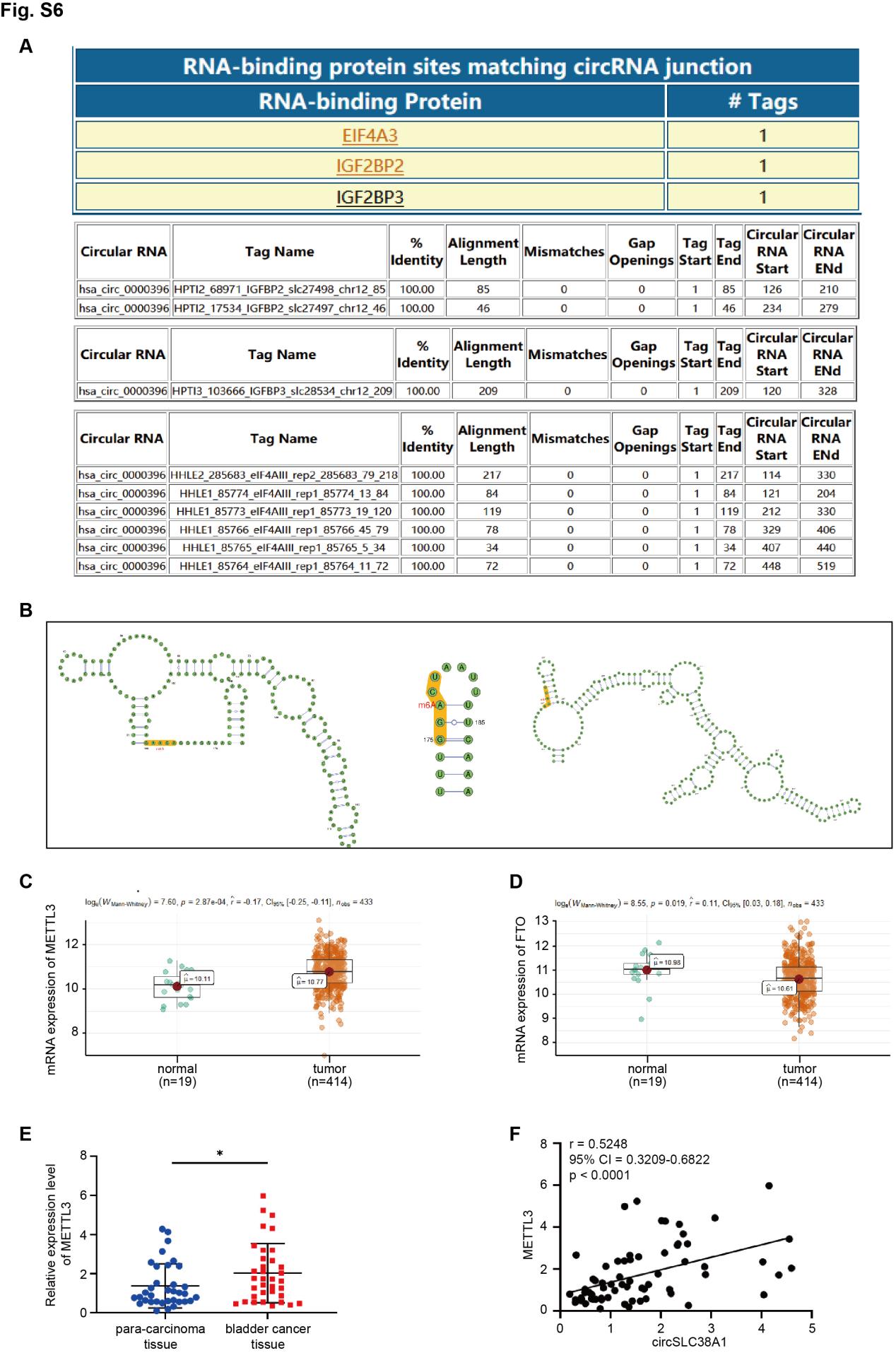
**

**Figure S6. m6A modification is involved in the upregulation of circSLC38A1 in BC cells.**

**a** Three m6A “reader’’ were found to bind circSLC38A1 at the junction site by using *CircInteractome.* **b** m6A modification sites on the circSLC38A1 sequence with high or very high confidence were predicted by using *SRAMP*. **c** m6A “writer” METTL3 was highly expressed in BC based on data available from TCGA database. **d** m6A “eraser” FTO was downregulated in BC based on data available from TCGA database. **e** METTL3 was highly expressed in BC tissues detected by qRT-PCR. **f** The correlation between the expression of METTL3 and circSLC38A1 in samples of Cohort I was analysed using Spearman correlation analysis.

**
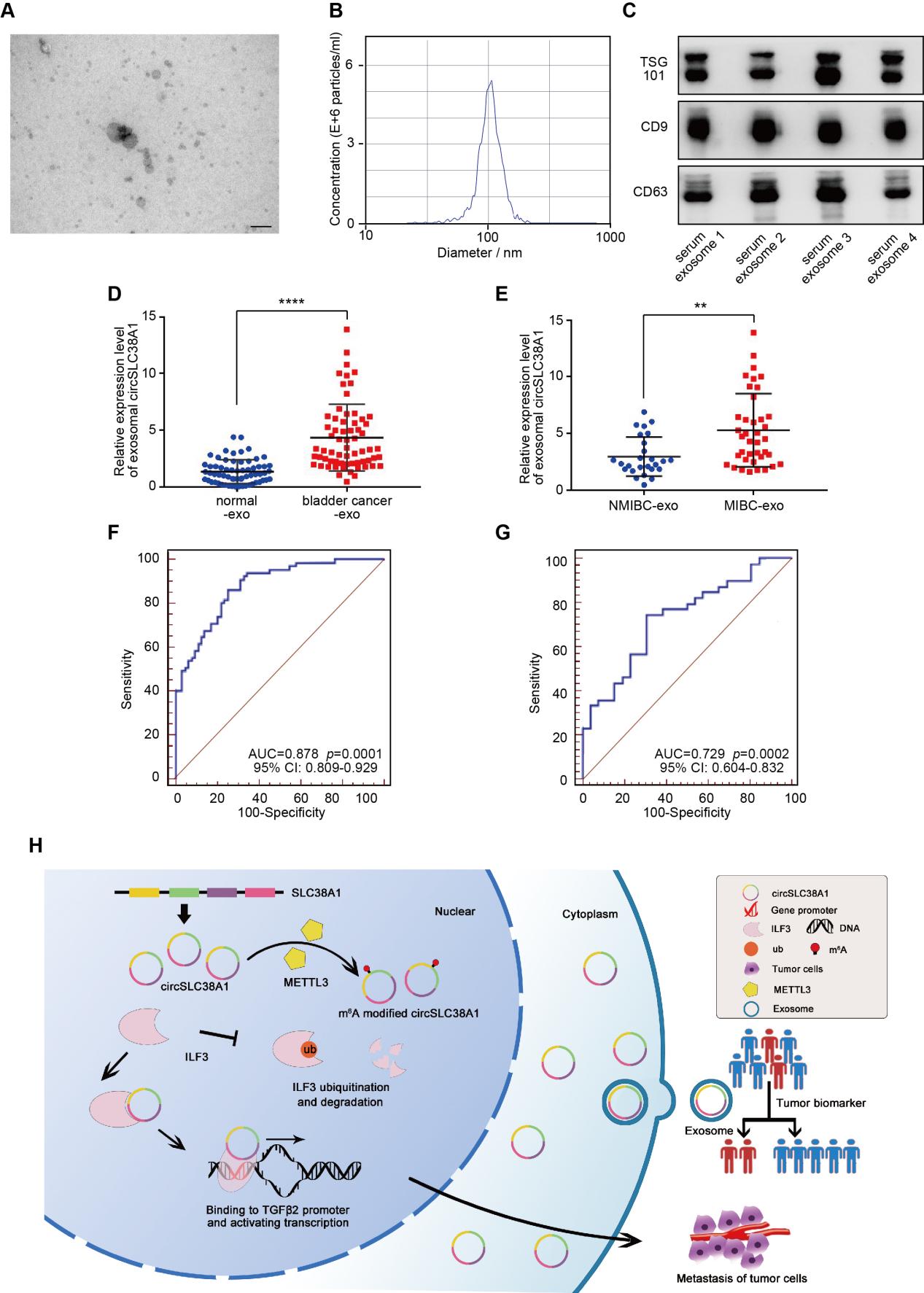
**

**Figure S7. Exosomal circSLC38A1 may act as a potential serum biomarker of BC. a** Identification of serum exosomes by TEM; scale bar: 100 nm. **b** Size distribution of serum exosome diameters measured by NTA. **c** Detection of TSG101, CD9 and CD63 protein expression of serum exosome by western blot. **d** circSLC38A1 expression in serum exosome from BC patients and from non-cancerous donors were detected by qRT-PCR. **e** circSLC38A1 expression in serum exosome from MIBC patients and from NMIBC patients were detected by qRT-PCR. **f** ROC curve analyses and AUC values for exosomal circSLC38A1 in BC patients and non-cancerous donors. **g** ROC curve analyses and AUC values for exosomal circSLC38A1 in MIBC patients and NMIBC patients. **h** Model for m6A modified circSLC38A1 mediated metastasis in BC. m6A methylation-caused dysregulated circSLC38A1 could promote metastasis of BC by binding to and stabilizing ILF3, which further initiated TGF-β2 expression. Moreover, circSLC38A1 can be packaged into exosomes, and the exosomal circSLC38A1 may be a promising biomarker for clinical detection of BC.

**Supplementary Tables**

**Table S1.** Characteristics of the study population in cohort I.

| **Variable** | **Patients**  **n=41** |
| --- | --- |
| **Age (years)** |  |
| median | 65 |
| **Sex** |  |
| Male | 32 (78.05%) |
| Female | 9 (21.95%) |
| **TNM stage** |  |
| I (NMIBC) | 14 (34.14%) |
| II-IV (MIBC) | 27 (65.86%) |
| **Tumor grade** |  |
| Low grade | 6 (14.63%) |
| High grade | 35 (85.37%) |
| **Lymph node metastasis** |  |
| Negative | 35 (85.37%) |
| Positive | 6 (14.63%) |

Abbreviations: BC: bladder cancer; MIBC: muscle invasive bladder cancer;

NMIBC: non-muscle invasive bladder cancer.

**Table S2.** Characteristics of the study population in cohort III.

| **Variable** | **BC patients**  **n=65** | **Healthy people**  **n=64** | ***P*-Value** |
| --- | --- | --- | --- |
| **Age (years)** |  |  | 0.79 |
| ≤65 | 35 (53.85%) | 33 (51.56%) |  |
| >65 | 30 (46.15%) | 31 (48.44%) |  |
| **Sex** |  |  | 0.96 |
| Male | 52 (80.00%) | 51 (79.69%) |  |
| Female | 13 (20.00%) | 13 (20.31%) |  |
| **TNM stage** |  |  |  |
| I (NMIBC) | 41 (63.08%) |  |  |
| II-IV (MIBC) | 24 (36.92%) |  |  |
| **Tumor grade** |  |  |  |
| Low grade | 23 (35.38%) |  |  |
| High grade | 42 (64.62%) |  |  |
| **Lymph node metastasis** |  |  |  |
| Negative | 63 (96.92%) |  |  |
| Positive | 2 (3.08%) |  |  |

Abbreviations: BC: bladder cancer; MIBC: muscle invasive bladder cancer; NMIBC: non-muscle invasive bladder cancer.

**Table S3. Detailed information of 5 patients used for RNA sequencing.**

| **Patient #** | **Age** | **Sex** | **Disease stage** |
| --- | --- | --- | --- |
| **Patient #1** | 69 | Male | MIBC (stage II) |
| **Patient #2** | 39 | Male | NMIBC (stage I) |
| **Patient #3** | 47 | Female | MIBC (stage II) |
| **Patient #4** | 64 | Male | MIBC (stage II) |
| **Patient #5** | 43 | Male | NMIBC (stage I) |

Abbreviations: BC: bladder cancer; MIBC: muscle invasive bladder cancer;

NMIBC: non-muscle invasive bladder cancer.

**Table S4. List of primers for qRT-PCR.**

| **Gene** | **Sequence (5’-3’)** |
| --- | --- |
| circSLC38A1 | F: TTTTGGGACTCGCCTTTGCC |
|  | R: AATGGAAGCTTGACACCCCTG |
| SLC38A1 | F: TCCCTGCATTGTTCCAGAGC |
|  | R: TGACGGGTGGCAAACAAATG |
| GAPDH | F: GCACCGTCAAGGCTGAGAAC |
|  | R: TGGTGAAGACGCCAGTGGA |
| ILF3 | F: CGAACTCCTACAACTCTCCCG |
|  | R: CCTTGGTATGAGGAGCCGC |
| TGF-β2 | F: GCGCTACATCGACAGCAAAG |
|  | R: TGCAGCAGGGACAGTGTAAG |
| E-cadherin | F: AGTACAACGACCCAACCCAA |
|  | R: GGCTGTGCCTTCCTACAGAC |
| N-cadherin | F: TCCTGCTTATCCTTGTGCTGA |
|  | R: AAAAGTTGTTTGGCCTGGCG |
| Vimentin | F: AGGCGAGGAGAGCAGGATTT |
|  | R: AGTGGGTATCAACCAGAGGGA |
| Snail | F: GACCCCAATCGGAAGCCTAA |
|  | R: AGGGCTGCTGGAAGGTAAAC |

Abbreviations: qRT-PCR: quantitative Reverse Transcript PCR

**Table S5. List of sequences for siRNAs and shRNAs**

| **siRNA** | **Sequence (5’-3’)** |
| --- | --- |
| Si-circSLC38A1#1 | sense: AUCCUACUUUUUCUAUAUUTT |
|  | antisense: AAUAUAGAAAAAGUAGGAUTT |
| Si-circSLC38A1#2 | sense: AAUCCUACUUUUUCUAUAUTT |
|  | antisense: AUAUAGAAAAAGUAGGAUUTT |
| Si-ILF3#1 | sense: CCUGUGUGAGAAAUCCAUUTT |
|  | antisense: AAUGGAUUUCUCACACAGGTT |
| Si-ILF3#2 | sense: GGAUGGACAGAAGUUCCAATT |
|  | antisense: UUGGAACUUCUGUCCAUCCTT |
| Si-ILF3#3 | sense: CCAACCAUGGAGGCUACAUTT |
|  | antisense: AUGUAGCCUCCAUGGUUGGTT |
| Si-METTL3#1 | sense: GCAAGAAUUCUGUGACUAUTT |
|  | antisense: AUAGUCACAGAAUUCUUGCTT |
| Si-METTL3#2 | sense:GGUGACUGCUCUUUCCUUATT |
|  | antisense: UAAGGAAAGAGCAGUCACCTT |
| Si-METTL3#3 | sense: GGUUGGUGUCAAAGGAAAUTT |
|  | antisense: AUUUCCUUUGACACCAACCTT |
| Si-FTO#1 | sense: GCAGCUGAAAUAUCCUAAATT |
|  | antisense: UUUAGGAUAUUUCAGCUGCTT |
| Si-FTO#2 | sense: GCCAGUGAAAGGGUCUAAUTT |
|  | antisense: AUUAGACCCUUUCACUGGCTT |
| Si-FTO#3 | sense: GUGGCAGUGUACAGUUAUATT |
|  | antisense: UAUAACUGUACACUGCCACTT |
| Si-NC | sense: UUCUCCGAACGUGUCACGUTT |
|  | antisense: ACGUGACACGUUCGGAGAATT |
| Sh-circSLC38A1#2 | AATCCTACTTTTTCTATAT |
| Sh-NC | TTCTCCGAACGTGTCACGT |

**Table S6. CircRNAs with high expression levels and potential functional roles**

| **circRNA Name** | **Chromosome** | **Start site** | **End site** | **Homogenous mRNA** |
| --- | --- | --- | --- | --- |
| circSLC38A1 | 12 | 46229153 | 46243314 | SLC38A1 |
| circBRCA1-1 | 17 | 43095846 | 43124115 | BRCA1 |
| circBRCA1-2 | 17 | 43067608 | 43074521 | BRCA1 |
| circAP1M2 | 19 | 10583606 | 10584070 | AP1M2 |
| circFANCD2-1 | 3 | 10064729 | 10088950 | FANCD2 |
| circFANCD2-2 | 3 | 10046580 | 10049505 | FANCD2 |
| circTIAM-1 | 21 | 31182421 | 31203012 | TIAM1 |
| circTIAM-2 | 21 | 31182421 | 31195305 | TIAM1 |
| circSKP2 | 5 | 36166519 | 36177292 | SKP2 |

**Table S7. List of top 10 proteins enriched by circSLC38A1 sense probe**

| **Accession** | **Description** | **Mass** | **Score** | **emPAI** |
| --- | --- | --- | --- | --- |
| P02768 | Serum albumin  OS=Homo sapiens OX=9606 GN=ALB PE=1 SV=2 | 71317 | 165 | 0.25 |
| Q12906 | Interleukin enhancer-binding factor 3  OS=Homo sapiens OX=9606 GN=ILF3 PE=1 SV=3 | 95678 | 160 | 0.66 |
| P04264 | Keratin, type II cytoskeletal 1  OS=Homo sapiens OX=9606 GN=KRT1 PE=1 SV=6 | 66170 | 156 | 0.34 |
| P08670 | Vimentin  OS=Homo sapiens OX=9606 GN=VIM PE=1 SV=4 | 53676 | 108 | 0.13 |
| P13645 | Keratin, type I cytoskeletal 10  OS=Homo sapiens OX=9606 GN=KRT10 PE=1 SV=6 | 59020 | 100 | 0.24 |
| Q00839 | Heterogeneous nuclear ribonucleoprotein U  OS=Homo sapiens OX=9606 GN=HNRNPU PE=1 SV=6 | 91269 | 89 | 0.04 |
| P60709 | Actin, cytoplasmic 1  OS=Homo sapiens OX=9606 GN=ACTB PE=1 SV=1 | 42052 | 89 | 0.16 |
| P06733 | Alpha-enolase  OS=Homo sapiens OX=9606 GN=ENO1 PE=1 SV=2 | 47481 | 88 | 0.14 |
| P35527 | Keratin, type I cytoskeletal 9  OS=Homo sapiens OX=9606 GN=KRT9 PE=1 SV=3 | 62255 | 86 | 0.17 |
| Q08211 | ATP-dependent RNA helicase A  OS=Homo sapiens OX=9606 GN=DHX9 PE=1 SV=4 | 142181 | 81 | 0.10 |

**Table S8. Functional enrichment analysis of the downregulated genes**

| **ID** | **Description** | ***P* value** | **Gene ID** |
| --- | --- | --- | --- |
| GO:0050679 | positive regulation of epithelial cell proliferation | 9.66E-06 | NOG/CCND1/WNT7A/ITGB3/  VEGFA/CCL5/NME2/EGR3/  NR4A3 |
| GO:0044321 | response to leptin | 1.24E-05 | CCND1/INHBB/EDN1/NR4A3 |
| GO:0050673 | epithelial cell proliferation | 3.25E-05 | NOG/CCND1/WNT7A/ITGB3/  VEGFA/EHF/CCL5/NME2/  LGR5/EGR3/NR4A3/TGFB2 |
| GO:0062044 | regulation of epithelial to mesenchymal transition | 4.38E-05 | NOG/TGFB2 |
| GO:1905006 | regulation of epithelial to mesenchymal transition involved in endocardial cushion formation | 4.38E-05 | NOG/TGFB2 |
| GO:0061082 | myeloid leukocyte cytokine production | 4.44E-05 | DDX58/CD74/NR4A3/TGFB2 |
