# Supplementary material for: Characterization of circSCL38A1 as a novel oncogene in bladder cancer via targeting ILF3/TGF-β2 signaling axis
Source: Cell Death Dis. 2023 Jan 25;14(1):59. doi: 10.1038/s41419-023-05598-2 (PMC9876890; doi:10.1038/s41419-023-05598-2)

Figure.1E

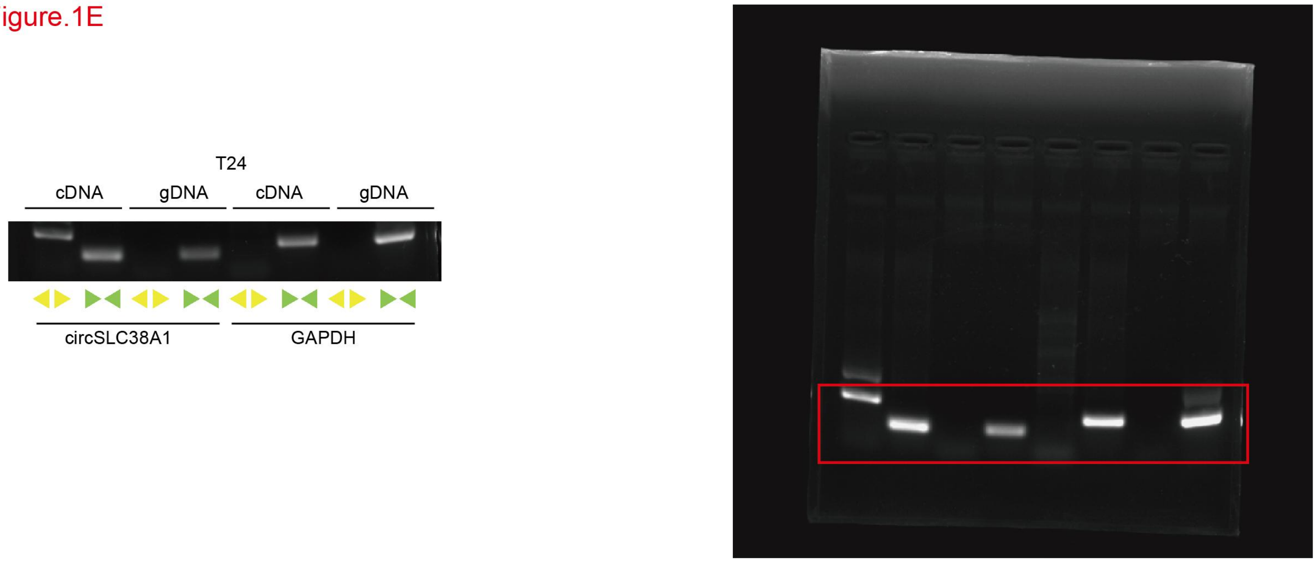

Figure.3I

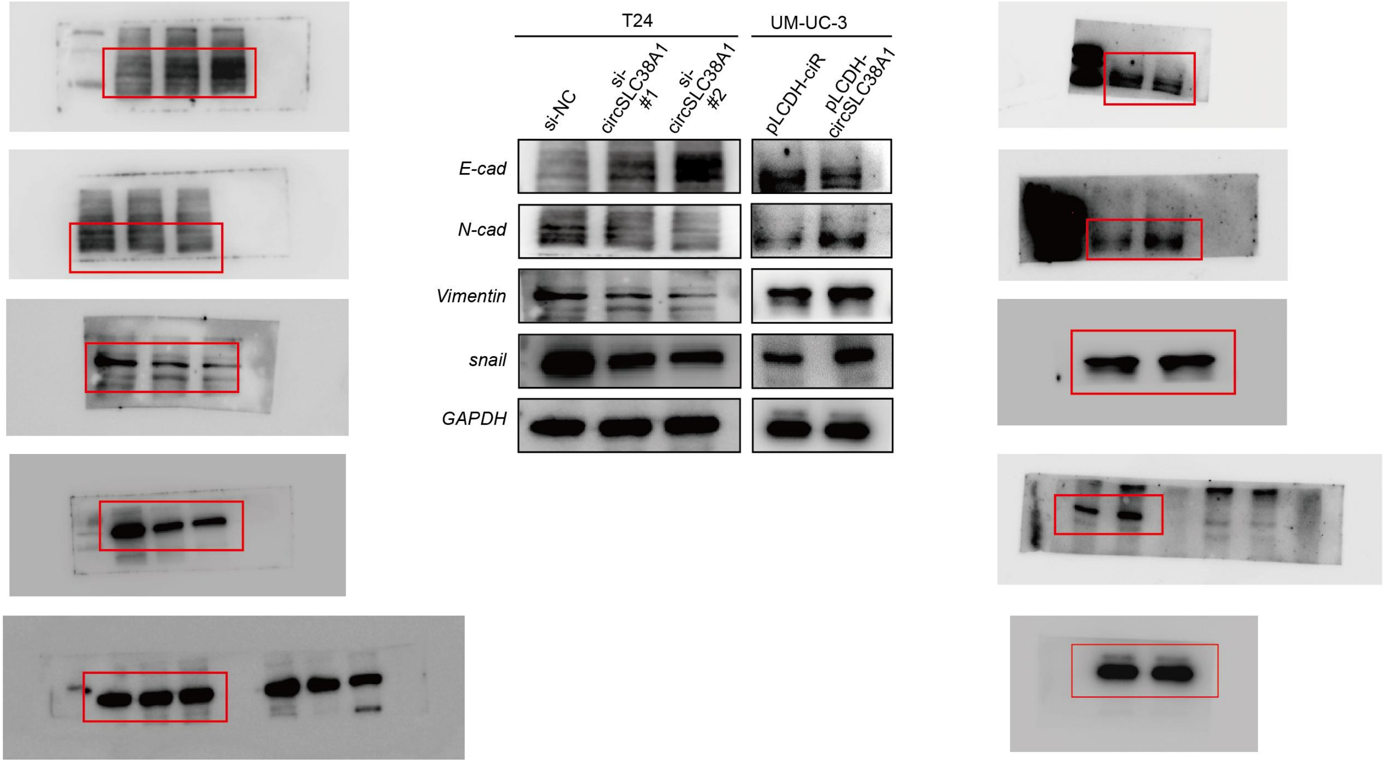

Figure.5A

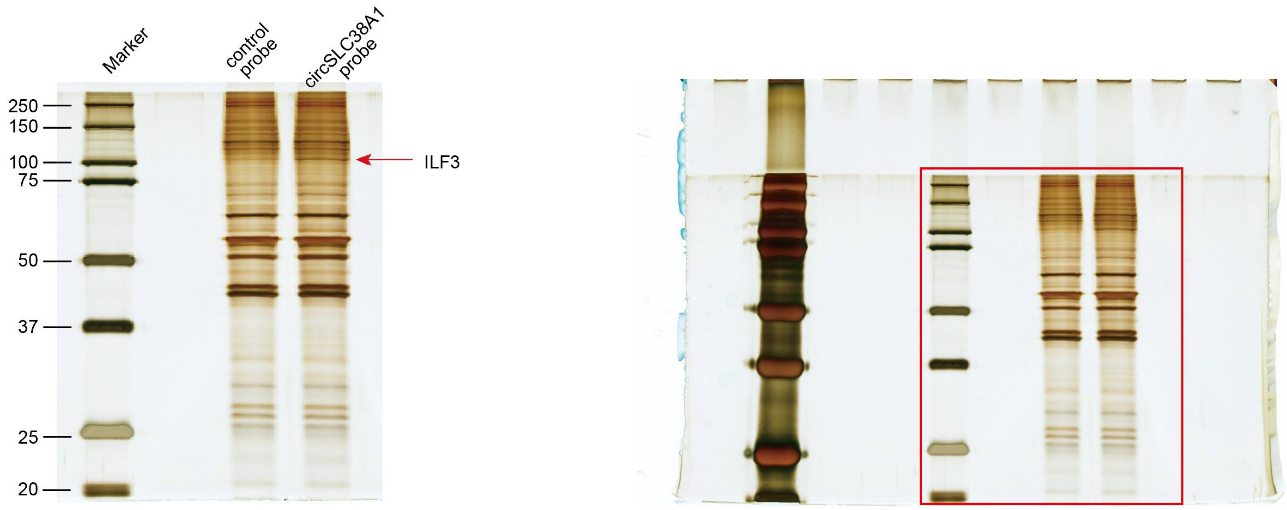

Figure.5B

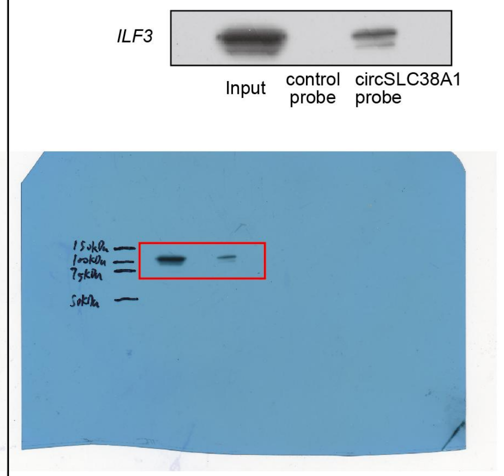

Figure.5C

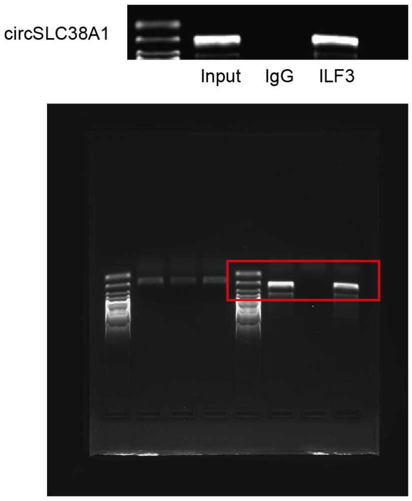

Figure.5F

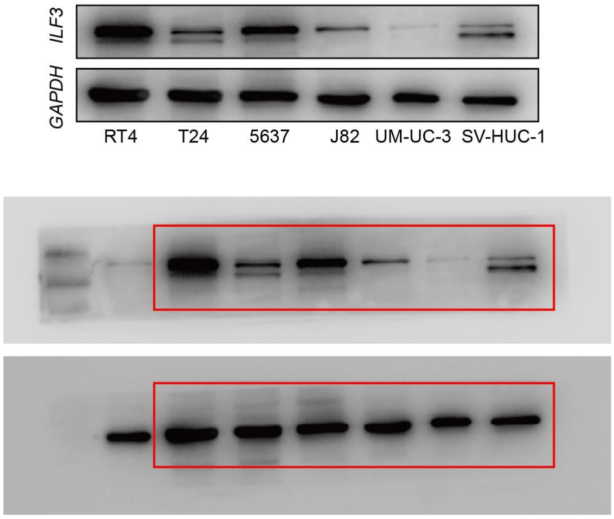

Figure.5J

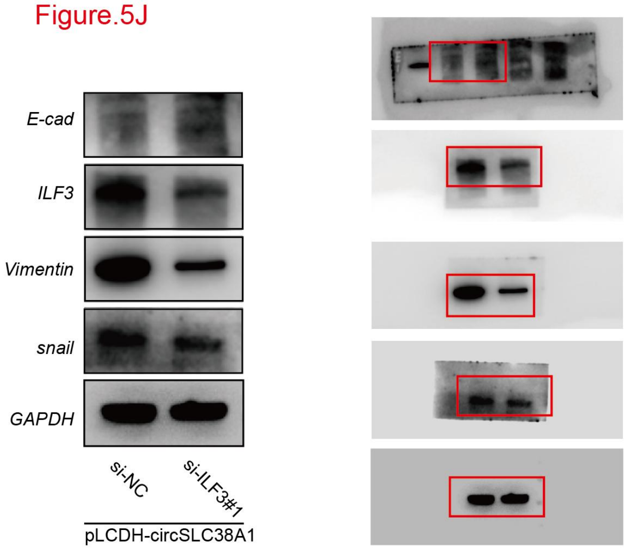

Figure.6A

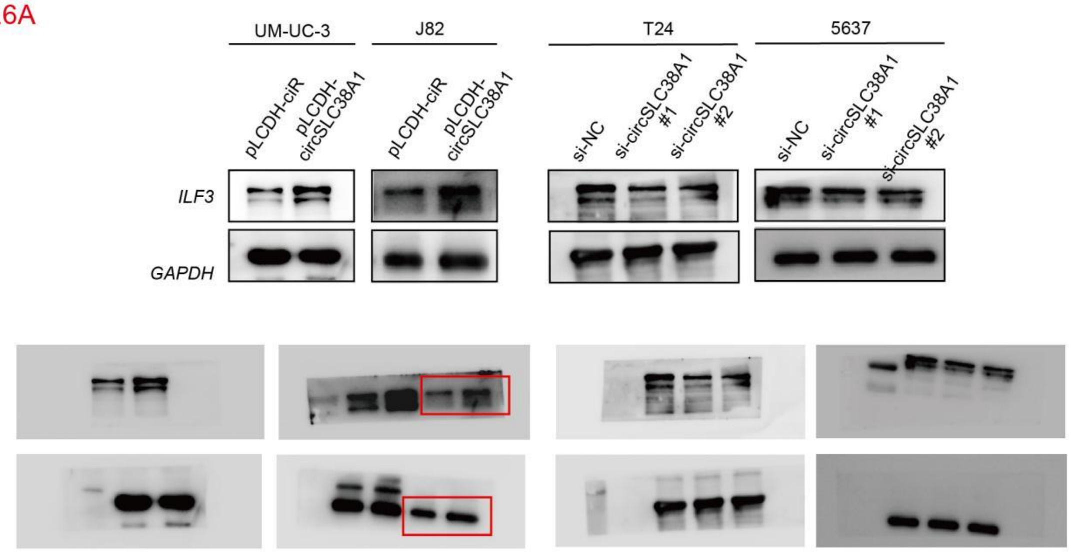

Figure.6B

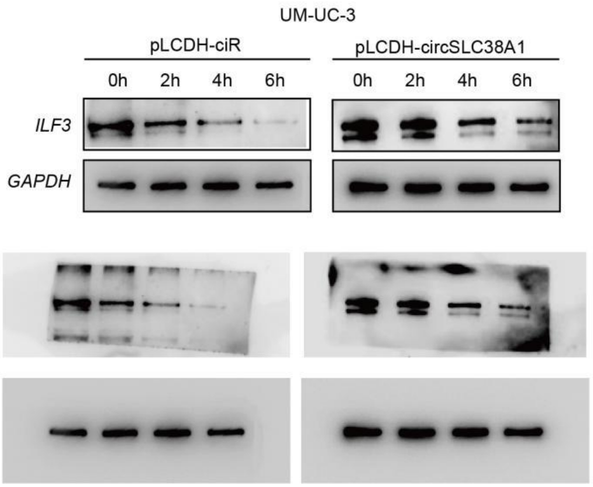

Figure.6C

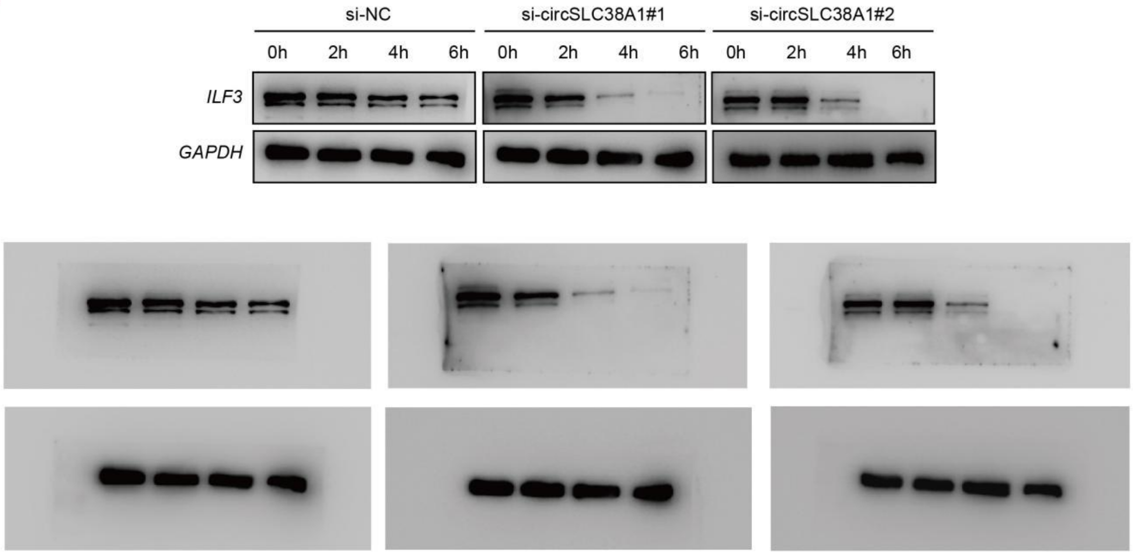

Figure.6D

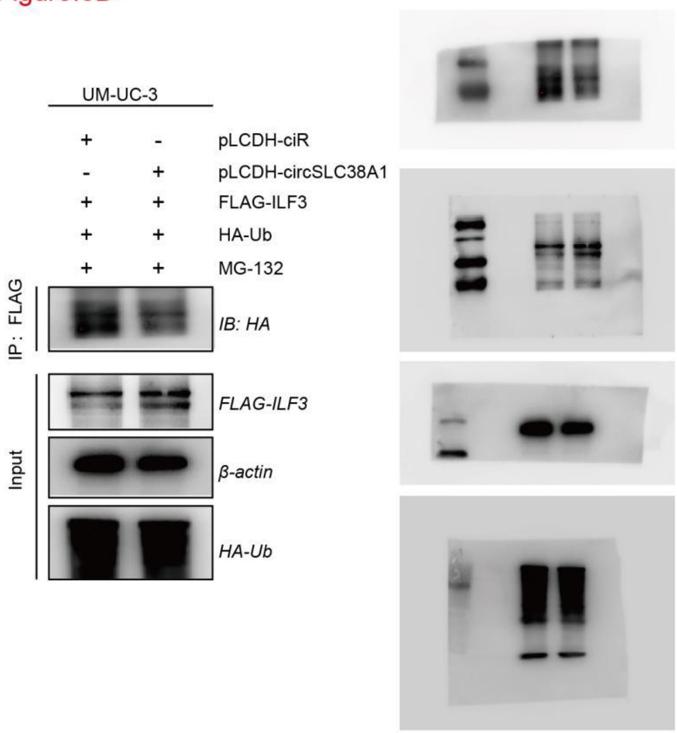

Figure.6E

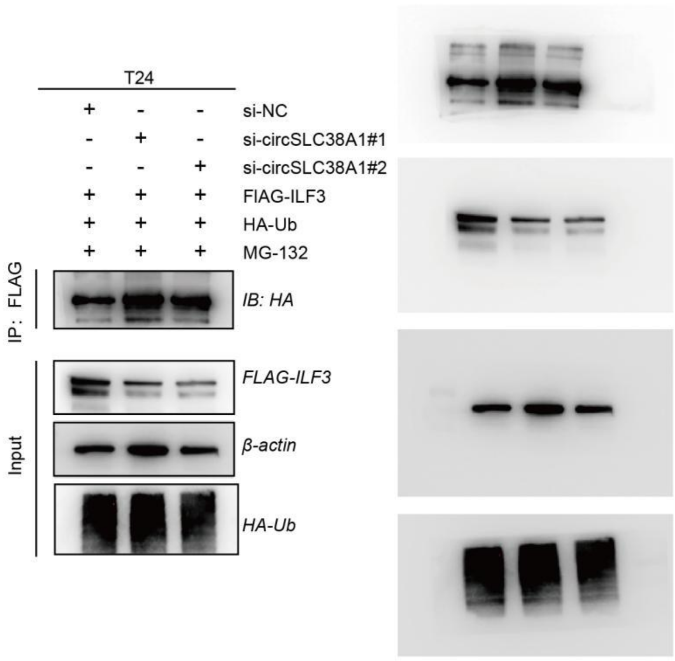

Figure.7F

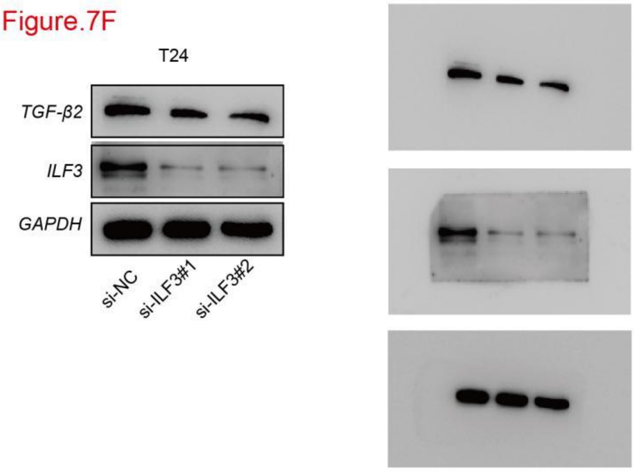

Figure.7H

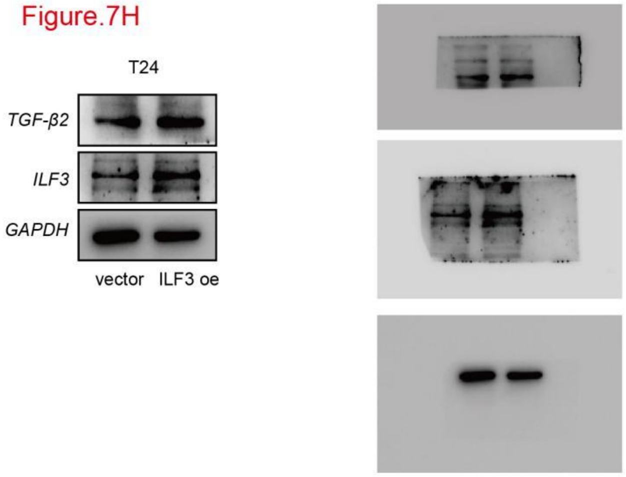

Figure.7J

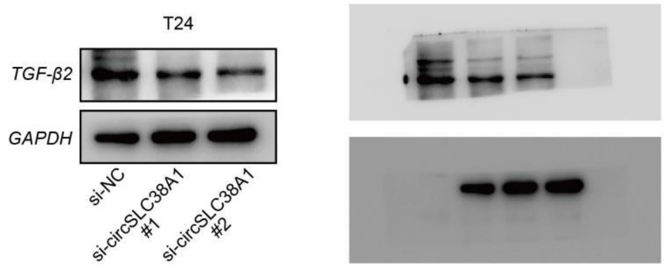

Figure.7L

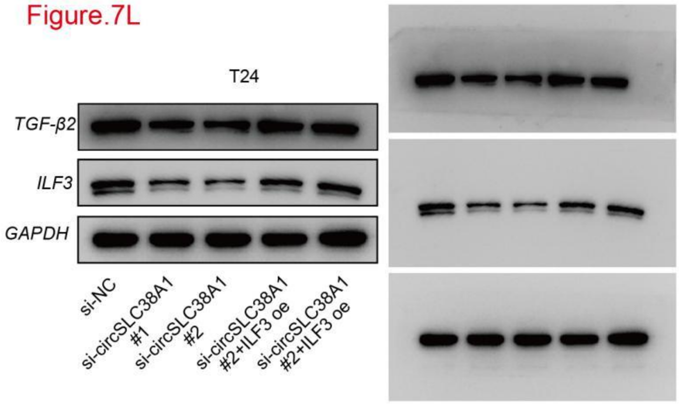

Figure.7M

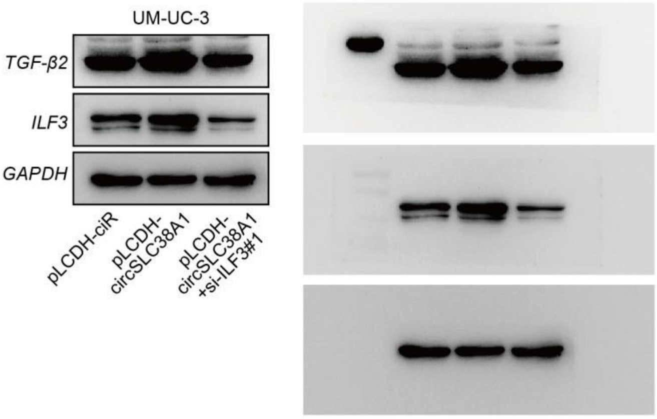

Figure.8D

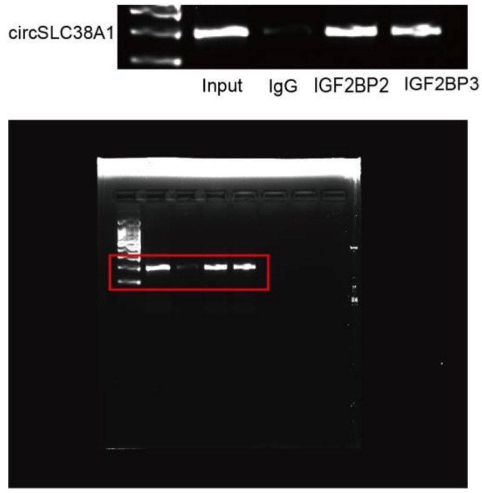

Figure.8G

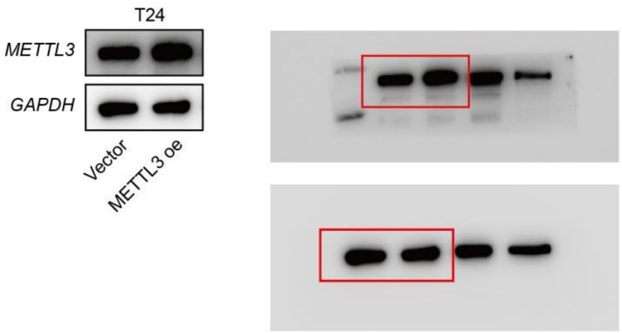

Figure.8H

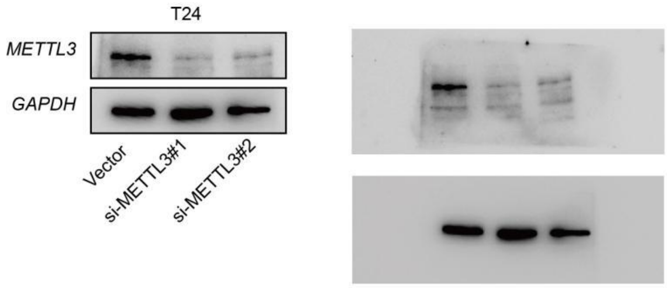

Figure.8I

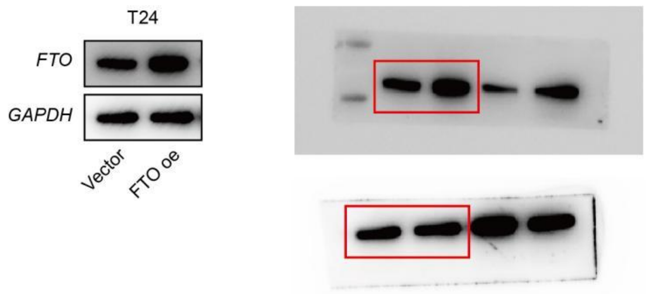

Figure.8J

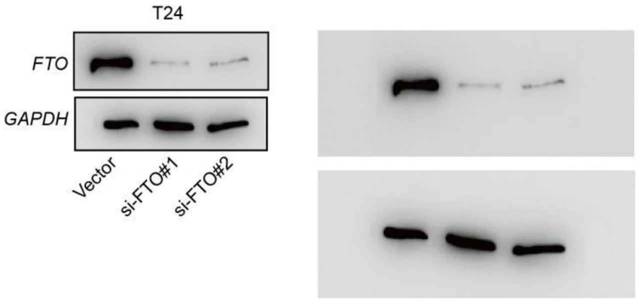

Figure.S2H

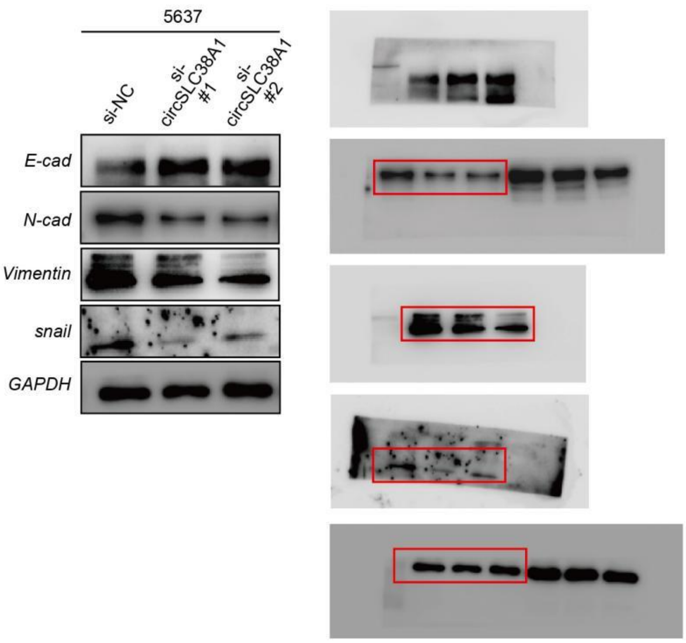

Figure.S2I

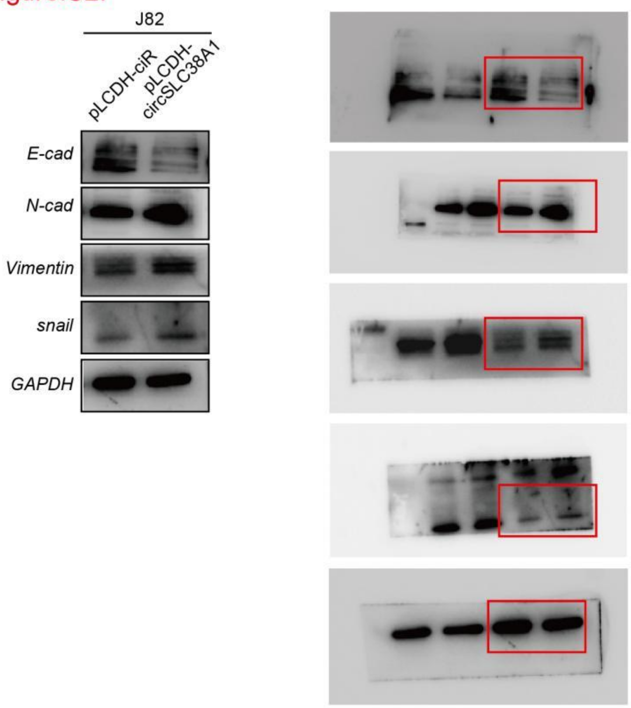

Figure.S4E

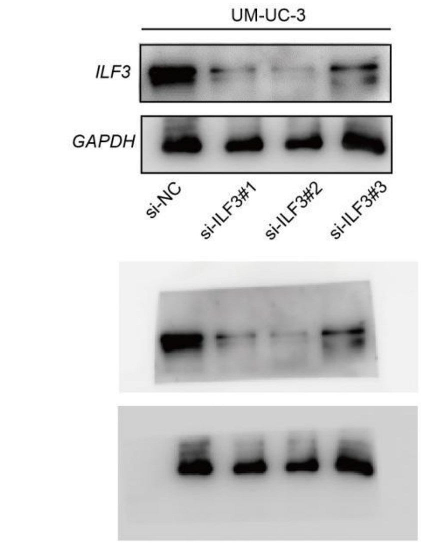

Figure.S7C

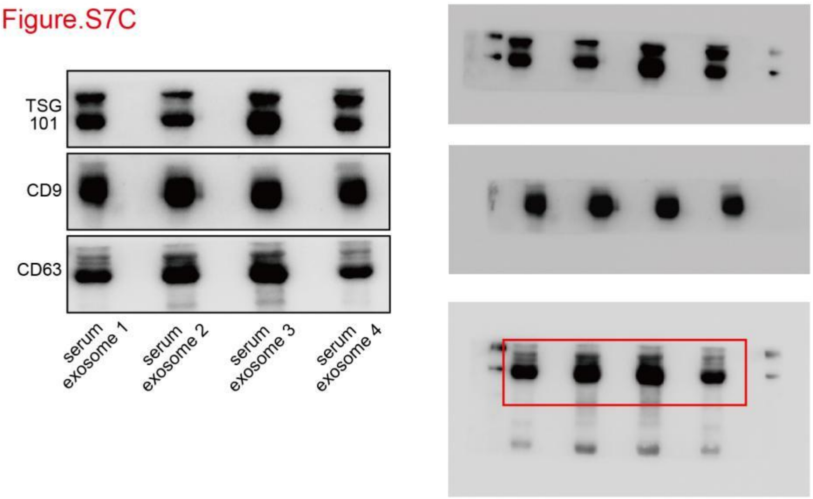

Supplement: Supplementary file 2 — Full and uncropped western blot [file 41419_2023_5598_MOESM2_ESM.pdf]
